# Water-mediated synthesis of hydrogen-bonded metal–organic frameworks†

**DOI:** 10.1039/d5sc02337h

**Published:** 2025-07-23

**Authors:** Zongjing Xiao, Pengfei Li, Beibei Sun, Xinrui Bao, Lei Gan, Huajun Yang

**Affiliations:** a Jiangsu Key Laboratory of Biomedical Materials, College of Chemistry and Materials Science, Nanjing Normal University Nanjing 210023 China huajunyang@nnu.edu.cn; b School of Environment, Nanjing Normal University Nanjing 210023 China

## Abstract

The integration of different types of frameworks into one would provide a unique opportunity for the development of crystalline frameworks that can combine the unique advantages of each. In this work, we successfully realize the integration of metal–organic frameworks (MOFs) and hydrogen-bonded organic frameworks (HOFs) through the synthesis of two hydrogen-bonded metal–organic frameworks, namely NNM-1(M) (M = Cu or Ni). The structures of NNM-1 are built on (4, 4)-squared coordinative metal–organic layers. Between the layers, two adjacent protonated carboxylic groups serve as both hydrogen donors and acceptors, leading to the formation of hydrogen-bonded pillars with *R*_2_^2^(8) dimeric hydrogen bonding, the most common hydrogen-bonded motif in HOFs. It was found that water plays a vital role in the structural transformation from 3D MOFs to H-MOFs here. Under a low pressure of 0.1 bar, the material NNM-1(Cu) can adsorb 4.84 mmol g^−1^ of NH_3_, making it a promising adsorbent for ammonia capture.

## Abstract 2

In this work, we have reported the creation of a new family of materials with the integration of MOFs and HOFs into H-MOFs through a water-mediated synthetic strategy.

## Graphical abstract

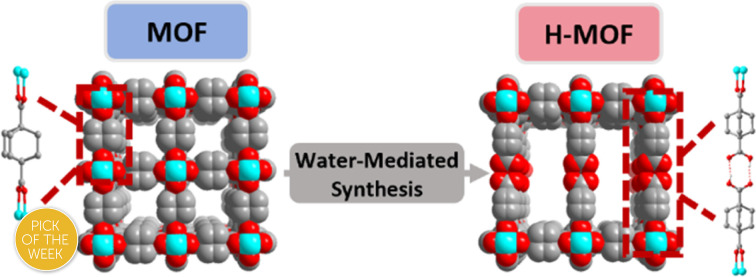

## 1 Introduction

Over the past several decades, crystalline porous materials, including metal–organic frameworks (MOFs),^1–4^ covalent organic frameworks (COFs),^5–7^ hydrogen-bonded organic frameworks (HOFs),^8,9^ and also some traditional porous materials, have experienced explosive growth.^10^ Each type of framework clearly has its own advantages that some or all of the others do not have. For example, HOFs, constructed by weak hydrogen bonding, have excellent self-healing and regeneration properties.^11^ COFs usually exhibit ultra-stable structures that can survive in very harsh chemical environments.^12^ In comparison with HOFs and COFs, the existence of metals endows MOFs with greater potential in gas adsorption and separation,^13^ catalysis,^14,15^ electrochemistry,^16–19^ and so on.

The integration of different types of frameworks into one should provide a unique opportunity for the development of crystalline frameworks that can combine the unique advantages of each. One such example is metal-covalent organic frameworks (or MCOFs),^20^ with the introduction of metal ions into COFs, which have emerged as a distinct class of porous materials with great potential in catalysis,^21^ optics,^22^ and so on.

We are interested in integrating MOFs and HOFs so that the new frameworks can inherit the advantages of both, such as the metal sites in MOFs and the excellent processability of HOFs. In fact, the coexistence of coordination bonds and hydrogen bonds in crystalline frameworks is not rare.^23–25^ However, in most scenarios, such frameworks would be simplified into a zero-dimensional structure based on coordination metal complexes or metal–organic cages without consideration of hydrogen bonding.^26,27^ In other words, the nature of the frameworks aligns with that of HOFs, which is also probably why such materials are commonly called M-HOFs.

In this work, we successfully realize the integration of metal–organic frameworks and hydrogen-bonded organic frameworks through the synthesis of two hydrogen-bonded metal–organic frameworks, namely NNM-1(M) (M = Cu or Ni). The structures of NNM-1 are built on (4, 4)-squared coordinative metal–organic layers with hydrogen-bonded pillars. Between the layers, two adjacent protonated carboxylic groups serve as both hydrogen donors and acceptors, leading to the formation of *R*_2_^2^(8) dimeric hydrogen bonding, the most common hydrogen-bonded motif in HOFs.

## 2 Results and discussion

### 2.1 Synthesis and structural features

Rod-shaped crystals (NNM-1) suitable for single crystal X-ray diffraction (SCXRD) can be obtained by a solvothermal reaction of M(NO_3_)_2_·*x*H_2_O (M = Cu or Ni), 1,4-diazabicyclo[2.2.2]octane (DABCO), and bicyclo[2.2.2]octane-1,4-dicarboxylic acid (H_2_BODC) in the mixed solvents of DMF and H_2_O. The phase purity of both crystals was validated using their PXRD patterns (a drop of HBF_4_ was required for NNM-1(Cu) to improve the phase purity, Fig. S1 and S2†). NNM-1 crystallized in the orthorhombic system with the space group of *Immm* (No. 71, Table S1†). The asymmetric unit contains one quarter of metal, one eighth of DABCO, one quarter of HBODC^−^, and one eighth of BODC^2−^, which thus gives a formula of M_2_(DABCO)(HBODC)_2_(BODC) (Fig. S3 and S4†). It is worth mentioning that the ligand of H_2_BODC has two kinds of deprotonated forms in the final structures including the fully deprotonated one (BODC^2−^) and the partially deprotonated HBODC^−^ with a protonated end. Two adjacent protonated carboxylic groups serve as both hydrogen donors and acceptors, leading to the formation of *R*_2_^2^(8) dimeric hydrogen bonding, the most common hydrogen-bonded motif in HOFs (Fig. S5†).^28^

**Fig. 1:**
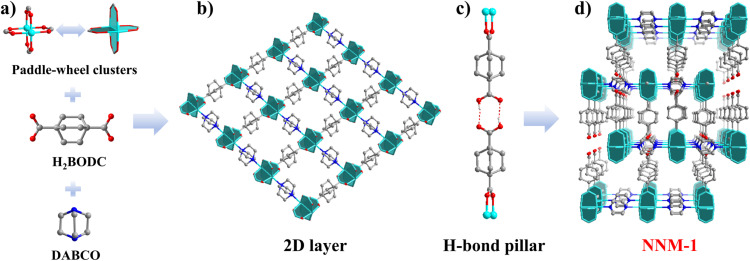
The assembly and structure of NNM-1: (a) three starting components for the assembly of NNM-1; (b) two-dimensional (4, 4)-squared layer; (c) hydrogen-bonded pillar (a pair of HBODC^−^); (d) double-interpenetrated structure of NNM-1. Color codes: Cu, sky blue; C, gray; H, white; O, red; and N, blue.

The structure of NNM-1 can be viewed as a pillar-layered structure with unique hydrogen-bonded pillars. The secondary building unit (SBU) of NNM-1 is the prototypical paddle-wheel metal dimer. The two metals in the SBU connect two BODC^2−^ and two DABCO to form (4, 4)-squared layers (Fig. 1b). The layers are further pillared by pairs of hydrogen-bonded HBODC^−^ (Fig. 1c) to form pcu-type H-MOFs. The final NNM-1 possesses a double-interpenetrated structure composed of two evenly staggered and independent sets of frameworks (Fig. 1d and S6†).

**Scheme 1:**
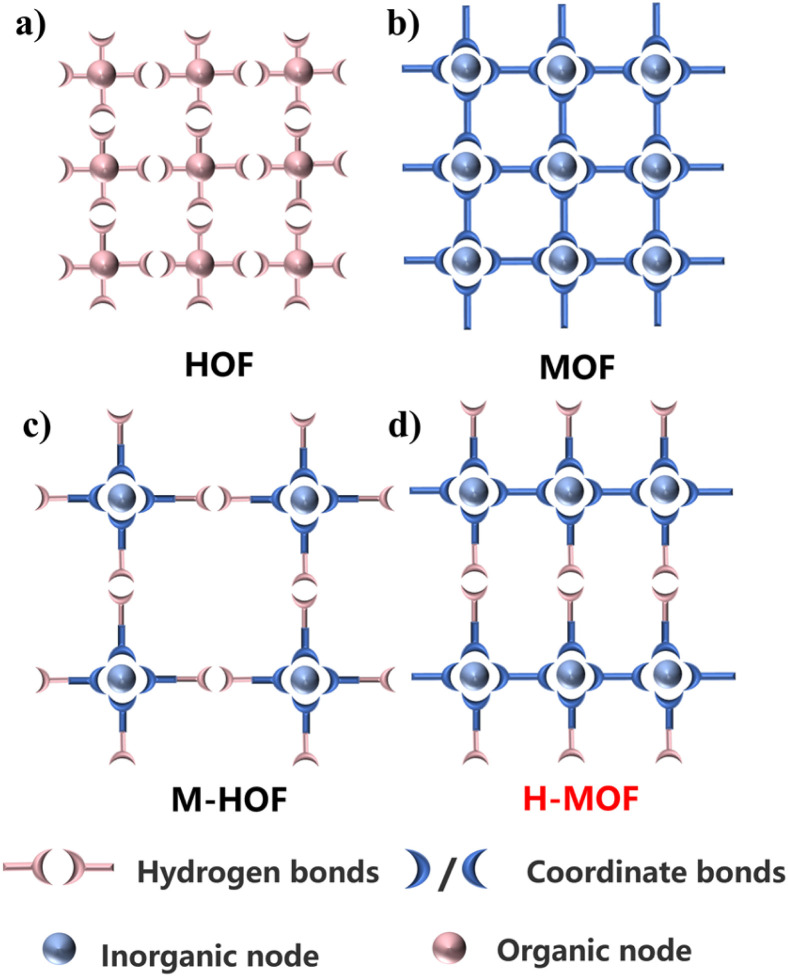
Schematic illustration of crystalline porous materials with different coordination modes. (a) Hydrogen-bonded Organic Frameworks (HOF); (b) Metal–Organic Frameworks (MOF); (c) Metal–Organic Hydrogen-Bonded Frameworks (M-HOF); (d) Hydrogen-Bonded Metal–Organic Frameworks (H-MOF).

The most striking structural feature of NNM-1 is the observation of the prototypical hydrogen-bonded motif as the linkage in a MOF structure. In fact, the coexistence of coordination bonds and hydrogen bonds in crystalline frameworks is not rare. However, in most scenarios, such frameworks would be simplified to a zero-dimensional structure based on coordination metal complexes or metal–organic cages without consideration of hydrogen bonding.^29,30^ In other words, the nature of the frameworks aligns with that of HOFs, which is also probably why such materials are commonly called M-HOFs (Scheme 1). NNM-1 is still two-dimensional and porous without considering hydrogen bonding, which still falls into the category of MOFs.

**Fig. 2:**
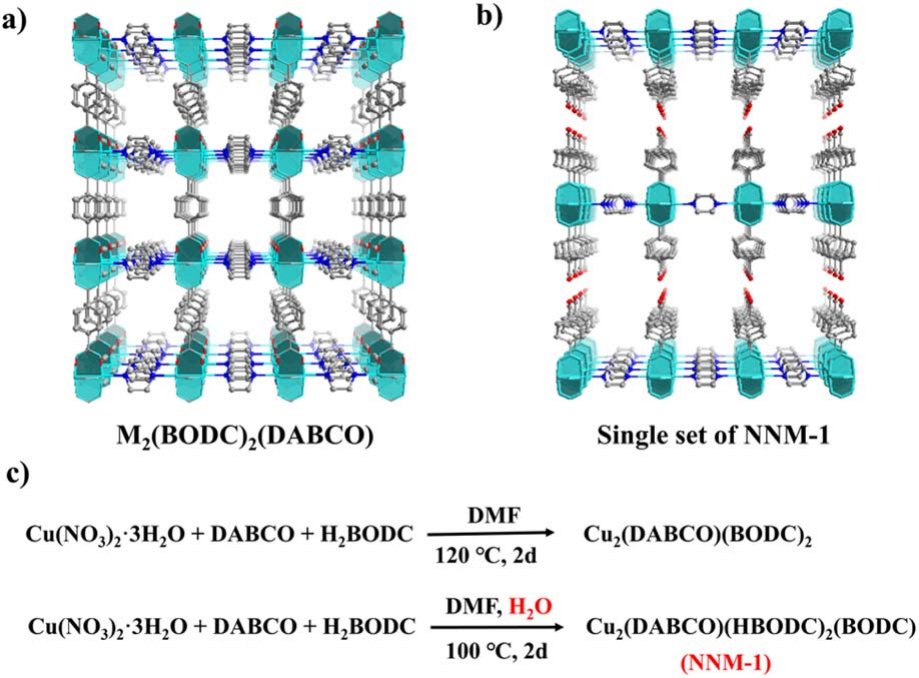
Comparison of the structures of (a) M_2_(BODC)_2_(DABCO) and (b) a single set of NNM-1; and (c) the reaction scheme for M_2_(BODC)_2_(DABCO) and NNM-1.

NNM-1 bears a close resemblance to the three-dimensional M_2_(BODC)_2_(DABCO) MOF. The transformation from M_2_(BODC)_2_(DABCO) to NNM-1 could be viewed as the replacement of one BODC^2−^ ligand in one direction by a pair of HBODC^−^ (Fig. 2). Alternatively, NNM-1 can also be treated as defective M_2_(BODC)_2_(DABCO) with missing-cluster defects.

It was found that water plays a vital role in the structural transformation from M_2_(BODC)_2_(DABCO) to NNM-1. For the synthesis of M_2_(BODC)_2_(DABCO), only DMF was used as the solvent.^31–33^ With the addition of water, the final product transformed to NNM-1, even with the same metal to ligand ratio (Fig. S7†). Such a synthetic rule was subsequently found to be applicable to a series of DMF/water solvents with different volume ratios and different reaction temperatures (Fig. S8–S11†). As shown in Fig. S10 and S11,† regardless of whether the reaction temperature is 100 °C or 120 °C, the product obtained is NNM-1 as long as the water is added, while the product is M_2_(BODC)_2_(DABCO) without the addition of water. A possible reason is that Cu^2+^/Ni^2+^, as a borderline Lewis acid (not too hard or too soft), has a relatively balanced affinity for water and all the ligands. Therefore, a large amount of water can compete with the ligands to coordinate with Cu^2+^/Ni^2+^, leading to missing-cluster defects in NNM-1.

**Fig. 3:**
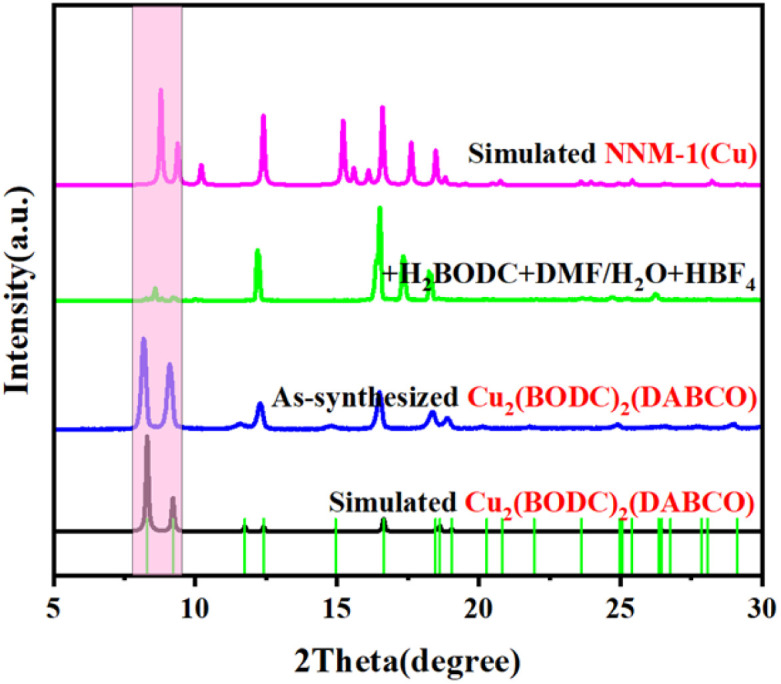
The variation of PXRD patterns during the post-synthesis of NNM-1(Cu) with Cu_2_(BODC)_2_(DABCO) as the raw material.

We also directly put Cu_2_(BODC)_2_(DABCO) crystals and H_2_BODC ligands into the solvents (DMF and water with 1 drop of HBF_4_) and after aging for 2 days at 100 °C, the cell parameters obtained by single crystal diffraction tests are identical to those of NNM-1(Cu), and the PXRD pattern agrees well with that of NNM-1(Cu), which can sufficiently prove that the final product has transformed into NNM-1(Cu) (Fig. 3).

### 2.2 Stability

As can be seen from the TGA curves of NNM-1(Cu), the structure gradually collapses at around 300 °C and completely collapses after 400 °C (Fig. S12†). NNM-1(Ni) has much higher thermal stability, which does not degrade until almost 450 °C (Fig. S13†). The chemical stability of both materials was also tested by immersing them in aqueous solutions of different pH for 1 day. PXRD patterns indicated that both materials could maintain the crystallinity in solutions with pH from 3 to 11, which was further confirmed by CO_2_ adsorption with essentially unchanged CO_2_ uptake (Fig. S14–S17†).

### 2.3 Porosity and NH_3_ adsorption isotherms

The guest-accessible volume was determined to be 167 Å^3^ per unit cell (∼8%) using the PLATON program. N_2_ adsorption isotherms at 77 K showed negligible uptake for both materials (Fig. S18 and S19†). The permanent porosity of the two MOFs was thus evaluated based on the CO_2_ adsorption isotherms collected at 195 K (Fig. S20 and S21†). The Brunauer–Emmett–Teller (BET) specific surface areas of NNM-1(Cu) and NNM-1(Ni) were determined to be 67.82 m^2^ g^−1^ and 178.98 m^2^ g^−1^, respectively.

**Fig. 4:**
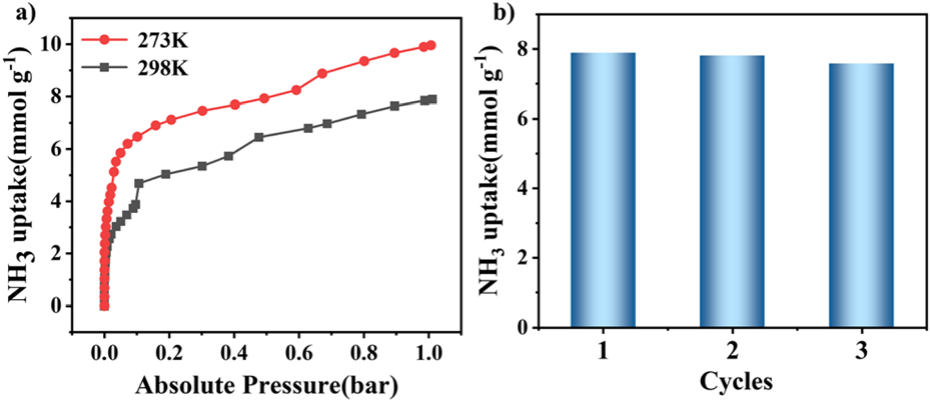
(a) NH_3_ adsorption isotherms of NNM-1(Cu) at 273 K and 298 K; (b) comparison of the maximum NH_3_ adsorption capacity of the three cycles of NNM-1(Cu) at 298 K.

NH_3_ capture properties were investigated due to the combination of relatively high stability, ultra-microporosity, and high density of carboxylic acid sites for NNM-1. Although NNM-1(Cu) has a lower surface area, its NH_3_ uptake at 1 bar is up to 7.89 mmol g^−1^ at 298 K and 9.96 mmol g^−1^ at 273 K, significantly higher than that of NNM-1(Ni) (Fig. 4a and S22). In particular, when converted to volumetric uptake, the uptake at 298 K is as high as 237.4 mg cm^−3^, higher than that of FDU–HOF–3, KUF-1a and other carboxylic acid-based frameworks (Table S2†).^34–36^ Under a low pressure of 0.1 bar, the material NNM-1(Cu) can adsorb 4.21 mmol g^−1^ of NH_3_ in the first cycle, and after two cycles, the amount of ammonia adsorbed in the third cycle at 0.1 bar even increased, reaching 4.84 mmol g^−1^ (Fig. S23–S26†). Under 25 mbar, NNM-1(Cu) can still adsorb 2.81 mmol g^−1^. As shown in Fig. 4b, the maximum adsorption capacity of NNM-1(Cu) remains essentially unchanged after three cycles, which indicates that this material possesses high cycling stability.

It could be reasonably proposed that NH_3_ uptake in NNM-1(Cu) occurs by chemisorption through breaking of the cupric carboxylate bond, as observed in Cu(cyhdc).^37^ Exposure of activated NNM-1(Cu) to ammonia solution also caused a color change from green to blue, confirming a significant change in the coordination environment of Cu^2+^ (Fig. S27†). The infrared spectrum (Fig. S28†) showed a distinct NH_4_^+^ vibration peak, indicating that NH_3_ reacted with –COOH in the structure of NNM-1 during the ammonia adsorption process. The PXRD patterns of NNM-1(Cu) after NH_3_ adsorption also showed a phase transformation with the main peaks shifting towards lower angles (Fig. S29 and S30†). Unfortunately, we failed to obtain the structure by single crystal XRD due to its low crystallinity.

In order to investigate the effect of NNM-1(Cu) on the removal of NH_3_ at low concentrations in air, we conducted breakthrough experiments for NH_3_ removal under dry conditions as well as at certain humidity (Fig. S31†) and obtained water adsorption curves for both materials (Fig. S32 and S33†). In the breakthrough test under dry conditions with a mixture of 1000 ppm ammonia in N_2_ through a fixed bed filled with NNM-1(Cu) at 298 K, a strong retention of ammonia was observed. As shown in the breakthrough curves displayed in Fig. S29,† the breakthrough time of NH_3_ under dry air conditions is measured to be 142 min g^−1^ for NNM-1(Cu) and the saturation NH_3_ uptake amount is found to be 5.45 mmol g^−1^. NNM-1(Cu) showed a significant decrease in the NH_3_ sorption capacity under humid conditions, indicating competitive adsorption between H_2_O and NH_3_ molecules of the active adsorption sites, even though the water adsorption capacity is relatively low in a wide pressure range.

## 3 Conclusions

In summary, this work reports the creation of a new family of materials with the integration of MOFs and HOFs into H-MOFs. H-MOFs here are different from previously reported M-HOFs, for which the frameworks would be simplified into zero-dimensional structures based on coordination metal complexes or metal–organic cages without consideration of hydrogen bonding. It was found that water played a vital role in the structure transformation from three-dimensional MOFs to H-MOFs. The water-mediated synthesis of NNM-1 offers a promising synthetic strategy with great potential for the development of new materials and applications.

## Author contributions

H. Y. and L. G. conceived and designed the research. Z. X., P. L., B. S., and X. B. performed the synthesis and measurements. H. Y. and Z. X. wrote the paper. L. G. and H. Y. discussed the results and revised the paper. All authors have given approval to the final version of the manuscript.

## Conflicts of interest

The authors declare no conflict of interest.

## Supplementary Material

SC-016-D5SC02337H-s001

SC-016-D5SC02337H-s002

## Data Availability

The data that support the findings of this study are available in the ESI† of this article.

## References

[cit1] Yaghi O. M., Li G., Li H. (1995). Selective binding and removal of guests in a microporous metal–organic framework. Nature.

[cit2] Li H., Eddaoudi M., O'Keeffe M., Yaghi O. M. (1999). Design and synthesis of an exceptionally stable and highly porous metal-organic framework. Nature.

[cit3] Zhou H.-C., Long J. R., Yaghi O. M. (2012). Introduction to Metal–Organic Frameworks. Chem. Rev..

[cit4] Schoedel A., Li M., Li D., O'Keeffe M., Yaghi O. M. (2016). Structures of Metal–Organic Frameworks with Rod Secondary Building Units. Chem. Rev..

[cit5] Côté A. P., Benin A. I., Ockwig N. W., O'Keeffe M., Matzger A. J., Yaghi O. M. (2005). Porous, Crystalline, Covalent Organic Frameworks. Science.

[cit6] Geng K., He T., Liu R., Dalapati S., Tan K. T., Li Z., Tao S., Gong Y., Jiang Q., Jiang D. (2020). Covalent Organic Frameworks: Design, Synthesis, and Functions. Chem. Rev..

[cit7] El-Kaderi H. M., Hunt J. R., Mendoza-Cortés J. L., Côté A. P., Taylor R. E., O'Keeffe M., Yaghi O. M. (2007). Designed Synthesis of 3D Covalent Organic Frameworks. Science.

[cit8] He Y., Xiang S., Chen B. (2011). A Microporous Hydrogen-Bonded Organic Framework for Highly Selective C_2_H_2_/C_2_H_4_ Separation at Ambient Temperature. J. Am. Chem. Soc..

[cit9] Hisaki I., Xin C., Takahashi K., Nakamura T. (2019). Designing Hydrogen-Bonded Organic Frameworks (HOFs) with Permanent Porosity. Angew. Chem., Int. Ed..

[cit10] Song J. H., Kang D. W. (2023). Hazardous nitroaromatic explosives detection by emerging porous solid sensors. Coord. Chem. Rev..

[cit11] Chen C., Shen L., Lin H., Zhao D., Li B., Chen B. (2024). Hydrogen-bonded organic frameworks for membrane separation. Chem. Soc. Rev..

[cit12] Zhang Z., Dong X., Yin J., Li Z. G., Li X., Zhang D., Pan T., Lei Q., Liu X., Xie Y., Shui F., Li J., Yi M., Yuan J., You Z., Zhang L., Chang J., Zhang H., Li W., Fang Q., Li B., Bu X. H., Han Y. (2022). Chemically Stable Guanidinium Covalent Organic Framework for the Efficient Capture of Low-Concentration Iodine at High Temperatures. J. Am. Chem. Soc..

[cit13] Senkovska I., Bon V., Mosberger A., Wang Y., Kaskel S. (2025). Adsorption and Separation by Flexible MOFs. Adv. Mater..

[cit14] Virender V., Pandey V., Singh G., Sharma P. K., Bhatia P., Solovev A. A., Mohan B. (2024). Hybrid Metal-Organic Frameworks (MOFs) for Various Catalysis Applications. Top. Curr. Chem..

[cit15] Cai L., Khanpour M., Yin Q., Wang Z.-Y., Fang Z.-B., Liu H.-X., Hou Y., Liu C., Deng W., Liu T.-F. (2024). Well-Defined Microenvironment in Metal–Organic Frameworks Enable Green, Benign, and Isolation-Free Catalytic Oxidation Reaction. CCS Chem..

[cit16] Ji L., Wang J., Wu K., Yang N. (2018). Tunable Electrochemistry of Electrosynthesized Copper Metal–Organic Frameworks. Adv. Funct. Mater..

[cit17] Yang L., He X., Dincă M. (2019). Triphenylene-Bridged Trinuclear Complexes of Cu: Models for Spin Interactions in Two-Dimensional Electrically Conductive Metal–Organic Frameworks. J. Am. Chem. Soc..

[cit18] Yang L., Dincă M. (2021). Redox Ladder of Ni3 Complexes with Closed-Shell, Mono-, and Diradical Triphenylene Units: Molecular Models for Conductive 2D MOFs. Angew. Chem., Int. Ed..

[cit19] Zhang A.-A. , Cheng X., He X., Liu W., Deng S., Cao R. and Liu T.-F., Harnessing Electrostatic Interactions for Enhanced Conductivity in Metal-Organic Frameworks, Research, 2021, 2021 10.34133/2021/9874273 PMC8556649 34778792

[cit20] Dong J., Han X., Liu Y., Li H., Cui Y. (2020). Metal-Covalent Organic Frameworks (MCOFs): A Bridge Between Metal-Organic Frameworks and Covalent Organic Frameworks. Angew Chem. Int. Ed. Engl..

[cit21] Han W. K., Liu Y., Feng J. D., Yan X., Pang H., Gu Z. G. (2023). Engineering a molecular ruthenium catalyst into three-dimensional metal covalent organic frameworks for efficient water oxidation. Chem. Sci..

[cit22] Liu T., Tao Q., Wang Y., Luo R., Ma J., Lei J. (2024). Tailored Cis-Trans Isomeric Metal-Covalent Organic Frameworks for Coordination Configuration-Dependent Electrochemiluminescence. J. Am. Chem. Soc..

[cit23] Wang X. Y., Liao S. Y., Huang H. P., Wang Q. F., Shi Y. Y., Zhu P. L., Hu Y. G., Sun R., Wan Y. J. (2023). Enhancing the Chemical Stability of MXene Through Synergy of Hydrogen Bond and Coordination Bond in Aqueous Solution. Small Methods.

[cit24] Guadalupe Vasquez-Rios M., Campillo-Alvarado G., MacGillivray L. R. (2023). Mechanochemical Mediated Coexistence of B<–N Coordination and Hydrogen Bonding. Angew Chem. Int. Ed. Engl..

[cit25] Yan Z., He M., Hu A., Liu M., Chen J., Liu J., Chen N., Cao L., Li B., Long J. (2023). Manipulating hydrogen and coordination bond chemistry for reversible zinc metal anodes. J. Colloid Interface Sci..

[cit26] Li H., Chao R., Liang J., Guo Z., Zhang B., Guo F. (2024). Metalo-Hydrogen-Bonded Organic Frameworks (MHOFs) Self-Assembled by Second-Sphere Coordination for Guest Exchange. Cryst. Growth Des..

[cit27] Zhu J.-Y., Kumari K., Chen S.-J., Shao D., Yang J., Shi L., Singh S. K., Zhang Y.-Z. (2024). Magnetic and Porous Regulation in Cobalt(II) Hydrogen-Bonded Organic Framework *via* Supramolecular Isomerism. Cryst. Growth Des..

[cit28] Li P., Ryder M. R., Stoddart J. F. (2020). Hydrogen-Bonded Organic Frameworks: A Rising Class of Porous Molecular Materials. Acc. Mater. Res..

[cit29] Zhu Z.-H., Wang H.-L., Zou H.-H., Liang F.-P. (2020). Metal hydrogen-bonded organic frameworks: structure and performance. Dalton Trans..

[cit30] Liu B.-T., Gong S.-H., Jiang X.-T., Zhang Y., Wang R., Chen Z., Zhang S., Kirlikovali K. O., Liu T.-F., Farha O. K., Cao R. (2023). A solution processible single-crystal porous organic polymer. Nat. Synth..

[cit31] Su Y. S., Lamb E. S., Liepuoniute I., Chronister A., Stanton A. L., Guzman P., Pérez-Estrada S., Chang T. Y., Houk K. N., Garcia-Garibay M. A., Brown S. E. (2021). Dipolar order in an amphidynamic crystalline metal–organic framework through reorienting linkers. Nat. Chem..

[cit32] Li K., Lee J., Olson D. H., Emge T. J., Bi W., Eibling M. J., Li J. (2008). Unique gas and hydrocarbon adsorption in a highly porous metal-organic framework made of extended aliphatic ligands. Chem. Commun..

[cit33] Zhou J., Wen K., Ke T., Li J., Jin Y., Li J., Zhang Z., Bao Z., Ren Q., Yang Q. (2024). Nonlinear 3D Ligand-Based Metal–Organic Framework for Thermodynamic–Kinetic Synergistic Splitting of Mono-/Dibranched Hexane Isomers. J. Am. Chem. Soc..

[cit34] Song X., Wang Y., Wang C., Gao X., Zhou Y., Chen B., Li P. (2023). Self-Healing Hydrogen-Bonded Organic Frameworks for Low-Concentration Ammonia Capture. J. Am. Chem. Soc..

[cit35] Kang D. W., Kang M., Kim H., Choe J. H., Kim D. W., Park J. R., Lee W. R., Moon D., Hong C. S. (2019). A Hydrogen-Bonded Organic Framework (HOF) with Type-IV NH_3_ Adsorption Behavior. Angew. Chem., Int. Ed..

[cit36] Ma K., Li P., Xin J. H., Chen Y., Chen Z., Goswami S., Liu X., Kato S., Chen H., Zhang X., Bai J., Wasson M. C., Maldonado R. R., Snurr R. Q., Farha O. K. (2020). Ultrastable Mesoporous Hydrogen-Bonded Organic Framework-Based Fiber Composites toward Mustard Gas Detoxification. Cell Rep. Phys. Sci.

[cit37] Snyder B. E. R., Turkiewicz A. B., Furukawa H., Paley M. V., Velasquez E. O., Dods M. N., Long J. R. (2023). A ligand insertion mechanism for cooperative NH3 capture in metal–organic frameworks. Nature.

