# Supplementary material for: Water-mediated synthesis of hydrogen-bonded metal–organic frameworks
Source: Chem Sci. 2025 Jul 23;16(33):14919–23. doi: 10.1039/d5sc02337h (PMC12284809; doi:10.1039/d5sc02337h)
Supplement: SC-016-D5SC02337H-s001 [file SC-016-D5SC02337H-s001.pdf]

Supplementary Information (SI) for Chemical Science.
This journal is © The Royal Society of Chemistry 2025

Water-Mediated Synthesis of Hydrogen-Bonded Metal-Organic Frameworks

Zongjing Xiao,[a] Pengfei Li,[b] Beibei Sun,[a] Xinrui Bao,[a] Lei Gan,[a] and Huajun Yang*[a]

Z. Xiao, B. Sun, X. Bao, Prof. L. Gan, Prof. H. Yang, Jiangsu Key Laboratory of Biomedical Materials, College of Chemistry and Materials Science, Nanjing Normal University, Nanjing 210023, China.

[b]P. Li, School of Environment, Nanjing Normal University, Nanjing 210023, China.

EXPERIMENTAL SECTION

Chemicals and Materials. Copper nitrate hexahydrate ($Cu(NO_3)_2 \cdot 3H_2O$, 99%), Nickel nitrate hexahydrate ($Ni(NO_3)_2 \cdot 6H_2O$, 99%), Bicyclo[2.2.2]octane-1,4-dicarboxylic acid (abbreviated as H_2BODC, 96%), 1,4-Diazabicyclo[2.2.2]octane (DABCO) and N,N-dimethylformamide (DMF, ≥ 99.8%). All chemicals were used without any further purification. All synthetic procedures were carried out in air unless noted otherwise.

Synthesis of NNM-1(Cu). 10.9 mg Copper nitrate trihydrate ($Cu(NO_3)_2 \cdot 3H_2O$, ~0.045 mmol), 7.1 mg H_2BODC (0.036mmol) and 3.3 mg DABCO (0.029mmol) were dissolved in 2 mL H_2O and 2 mL DMF, and then a drop of HBF_4 was added. After sonicated until both completely dissolved, the vial was placed in a 100 °C oven for 2 days, and the mixture was then cooled to room temperature. Green rod-like crystals were obtained. The phase purity was supported by powder X-ray diffraction.

Synthesis of NNM-1(Ni). 13 mg Nickel nitrate trihydrate ($Ni(NO_3)_2 \cdot 6H_2O$, ~0.045 mmol), 17.8 mg H_2BODC (0.09 mmol) and 5 mg DABCO (0.045 mmol) were dissolved in 2 mL H_2O and 2 mL DMF. After sonicated until both completely dissolved, the vial was placed in a 100 °C oven for 2 days and the mixture was then cooled to room temperature.

Green rod-like crystals were obtained. The phase purity was supported by powder X-ray diffraction.

Synthesis of $Cu_2(BODC)_2(DABCO)$. $Cu(NO_3)_2 \cdot 3H_2O$ (0.90 mmol), H_2BODC (0.72 mmol), and ted (0.58 mmol) were dissolved in 10 mL N,N-dimethylformamide (DMF). After sonicated, the vial was placed in a 120 °C oven for 2 days, and the mixture was then cooled to room temperature.

Single-Crystal X-ray Diffraction Characterization. The single-crystal X-ray diffraction measurements were performed on a Bruker APEX-II CCD diffractometer with graphitemonochromated Mo Kα radiation (λ = 0.71073 Å) at 296 K. Diffraction data were integrated and scaled by '\f and \w scans' method with the Bruker APEX software. The structure was solved by intrinsic phasing which was embedded in 'APEX Ⅲ' software and the refinement against all reflections of the compound was performed using 'APEX Ⅱ'. All non-hydrogen framework atoms were refined anisotropically. All the hydrogen atoms were calculated. CCDC 2433919-2433920 contain the supplementary crystallographic data for this paper. These data are provided free of charge by the Cambridge Crystallographic Data Centre.

Powder X-ray Diffraction (PXRD) Characterization. The crystallinity of the synthesized products was carried out by X-ray powder diffraction

(XRD) on D/max-rC X-ray diffractometer with Cu Kα radiation (λ = 1.5406 Å). The data collection was performed at room temperature in the range from 5° to 30° with a step size of ~0.01°. The simulated powder pattern was obtained from the single crystal data.

Thermogravimetric (TG) Measurement. Thermogravimetric (TGA) analysis was carried out on a HENVEN HCT-1 analyzer heated from ambient temperature to 800 °C under a nitrogen gas atmosphere with a heating rate of 10 °C/min.

FT-Infrared Experiments. Fourier transform infrared spectroscopy (FTIR) were recorded on a Brucker Tensor 27 in the range of 4000-400 cm^{-1} using the KBr pellets.

Gas Sorption Measurement. Gas sorption measurements were carried out on Automatic High Performance Surface Area and Aperture Analyzer (BSD-660 A3M). Prior to the measurement, the as-synthesized sample was purified by DMF and immersed in methanol solution for three days. During each day, the solution was refreshed. After solvent exchange, the upper solvent was decanted. The sample was first dried under air flow gently and was subsequently transferred into the test tube. The degas process was

performed under room temperature for 2 hours and further dried at 120 °C for another 10 hours.

Isosteric Heat of Adsorption (Qst). The isosteric heats of adsorption for NH_3 were calculated using the isotherms at 273 K and 298 K, following the Clausius-Clapeyron equation.

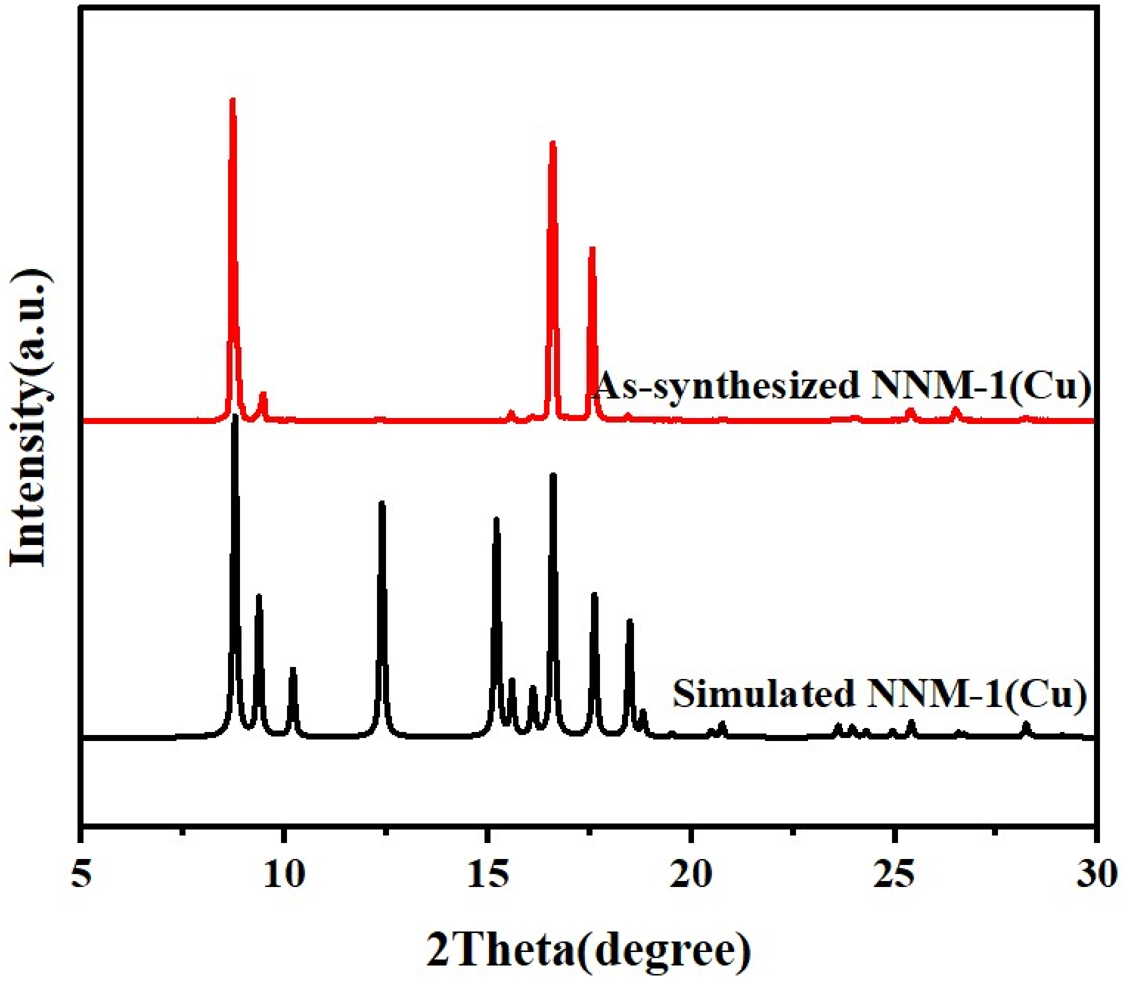

Figure S1. Comparison of simulated and as-synthesized PXRD patterns of **NNM-1(Cu)**.

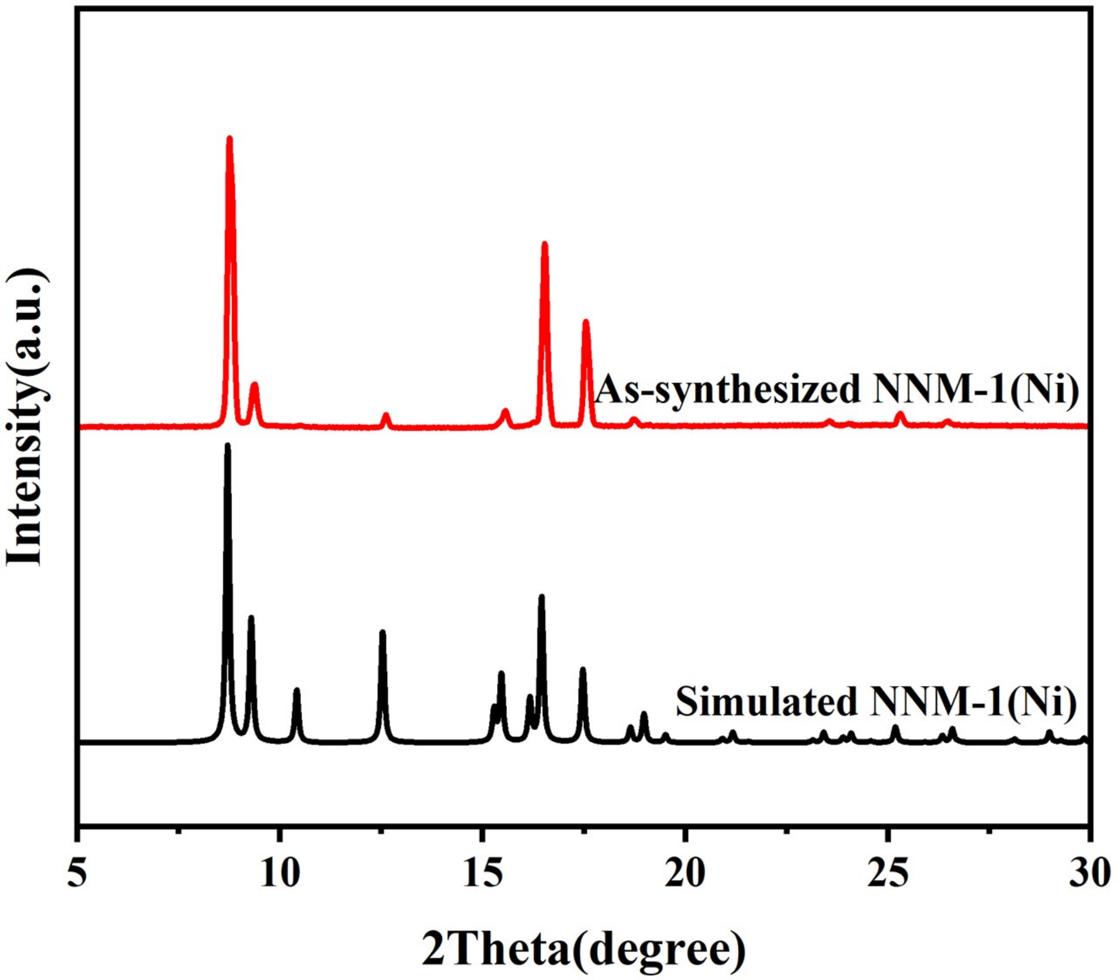

Figure S2. Comparison of simulated and as-synthesized PXRD patterns of **NNM-1(Ni)**.

Table S1. Crystallographic Data for **NNM-1(Cu) and NNM-1(Ni)**

Compound	**NNM-1(Cu)**	**NNM-1(Ni)**
chemical formula	$C_{36}H_{50}N_2O_{12}Cu_2$	$C_{36}H_{50}N_2O_{12}Ni_2$
formula weight	829.86	820.20
crystal size (mm)	0.15×0.10×0.05	0.18×0.15×0.10
temperature (K)	296(2)	296(2)
radiation	0.71073	0.71073
crystal system	orthorhombic	orthorhombic
space group	I m m m	I m m m
CCDC ref	2433919	2433920
a(Å)	9.610(3)	9.339(2)
b(Å)	10.687(4)	10.761(3)
c(Å)	20.153(7)	20.275(5)
α(°)	90.00	90.00
β(°)	90.00	90.00
γ(°)	90.00	90.00
V(Å^3)	2069.75(120)	2037.6(9)
Z	2	2
$\rho_{(calc)}$ (g/cm^3)	1.332	1.337
F (000)	868	864
absorp.coeff. (mm^{-1})	1.086	0.983
θ range (deg)	2.021 to 27.003	2.009 to 25.872
reflns collected	13114 (R_{int} = 0.0182)	12537(R_{int} = 0.0371)
indep. reflns	1265	1140
Refns obs. [$I>2\sigma(I)$]	1208	1055
data/restr/paras	1265/143/244	1140/137/162
GOF	1.015	0.949
R_1/wR_2 [$I>2\sigma(I)$]	0.0211/0.0597	0.0225/ 0.0581
R_1/wR_2 (all data)	0.0228/0.0613	0.0263/0.0604
larg peak and hole(e/Å^3)	0.374/-0.230	0.325/-0.209

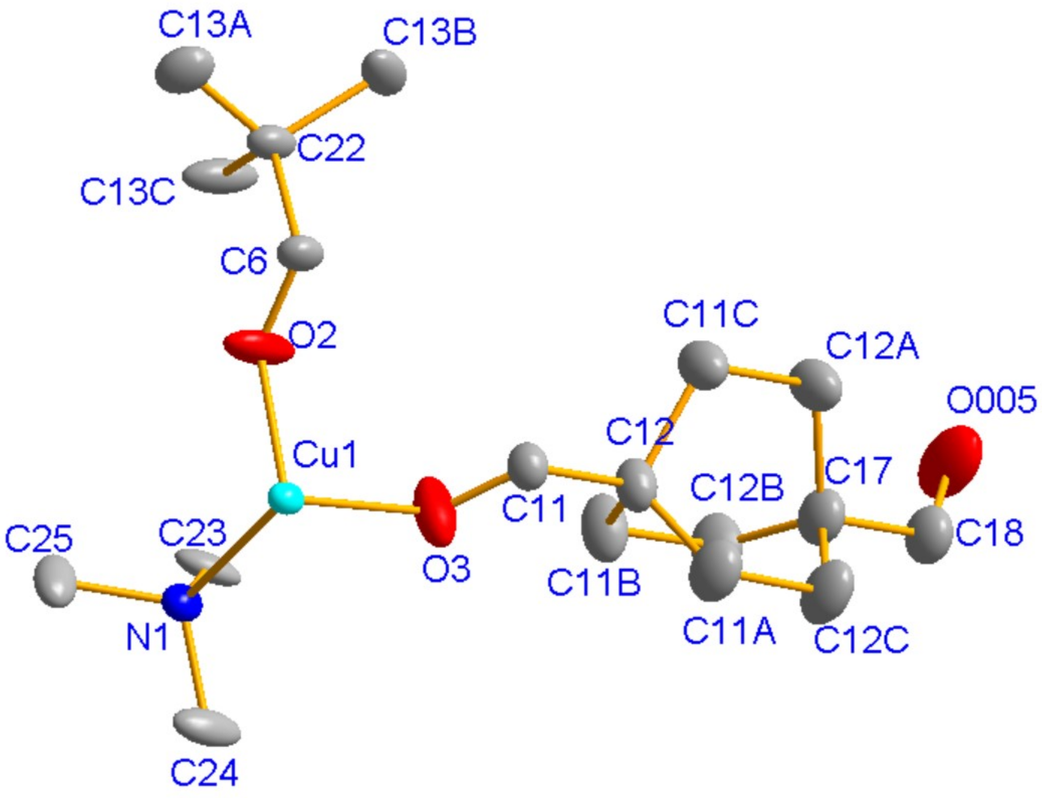

Figure S3. ORTEP drawing of the asymmetric unit of **NNM-1(Cu)** with 50% probability. (Blue: Cu; Red: Oxygen; Dark blue: Nitrogen; Gray: Carbon. Hydrogen atoms are omitted for clarity).

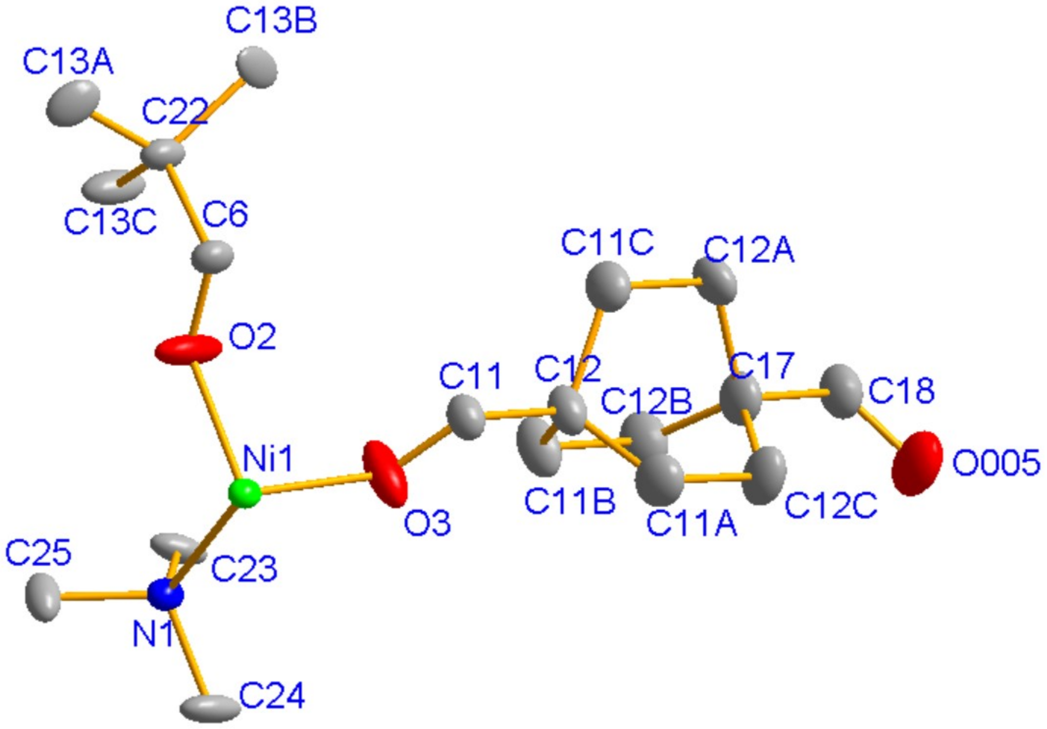

Figure S4. ORTEP drawing of the asymmetric unit of **NNM-1(Ni)** with 50% probability. (Green: Ni; Red: Oxygen; Dark blue: Nitrogen; Gray: Carbon. Hydrogen atoms are omitted for clarity).

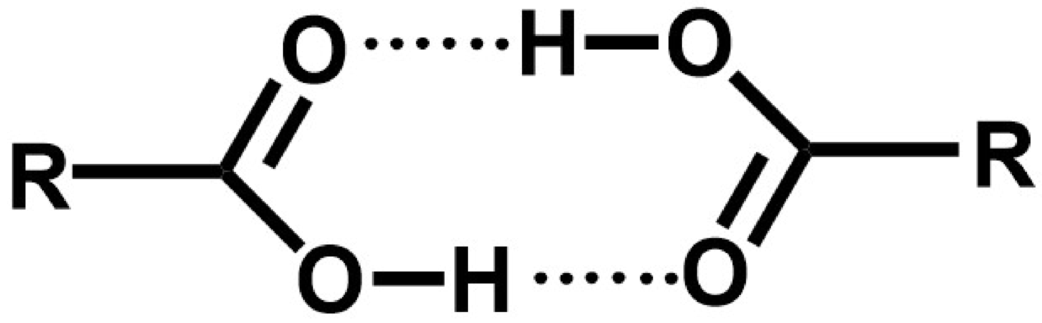

Figure S5. Schematic representation of the hydrogen-bonded **R2 2(8) homodimer** of the carboxyl group.

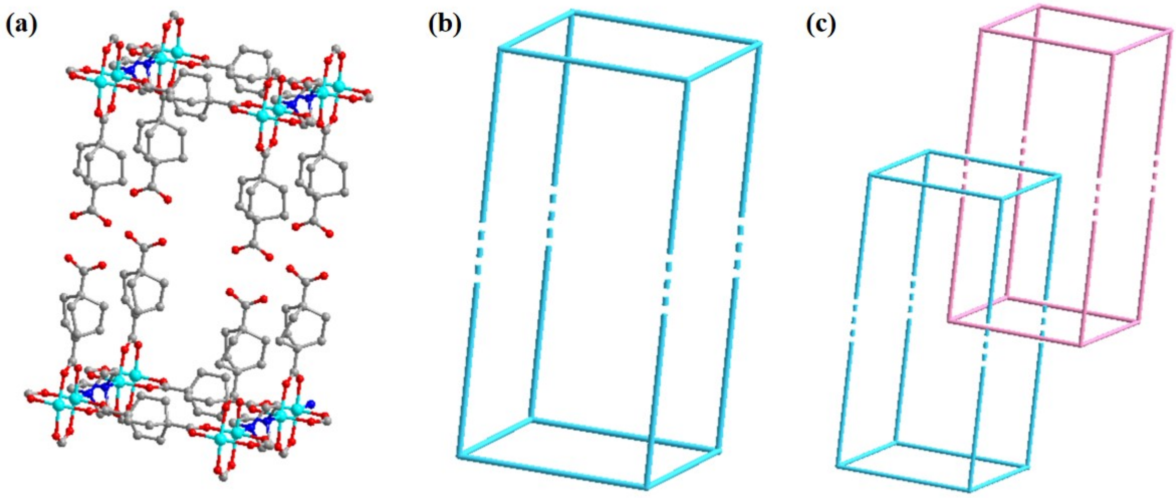

Figure S6. Illustration of the structure of **NNM-1(Cu)**. (a) Single pillar-layered 3D net; (b) Simplified single pillar-layered 3D net; (c) Twofold interpenetrating of the 3D net in **NNM-1(Cu)**.

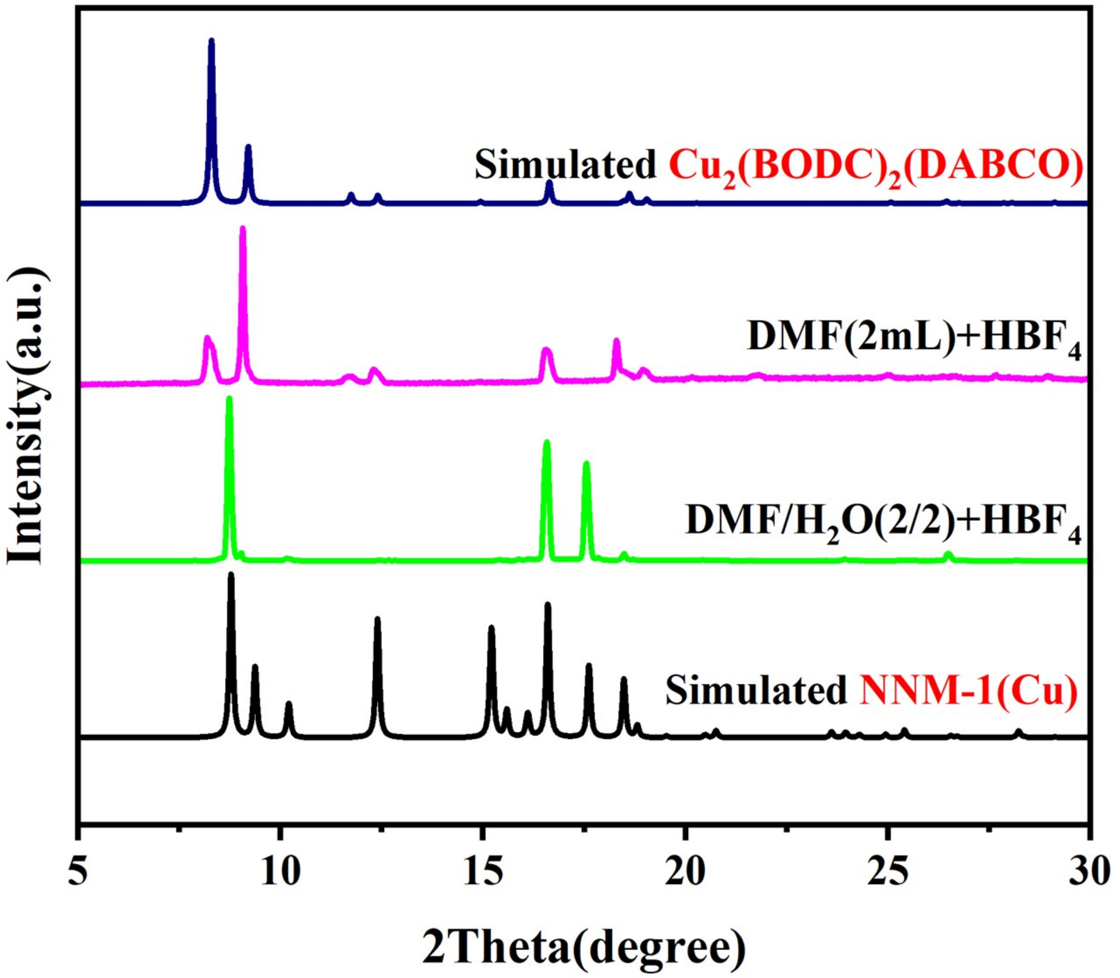

Figure S7. XRD comparisons of samples obtained by varying the solvent ratio of DMF/H_2O according to metal to ligands ratio in **NNM-1(Cu)** (that is Cu: BODC: DABCO = 0.045: 0.036: 0.029).

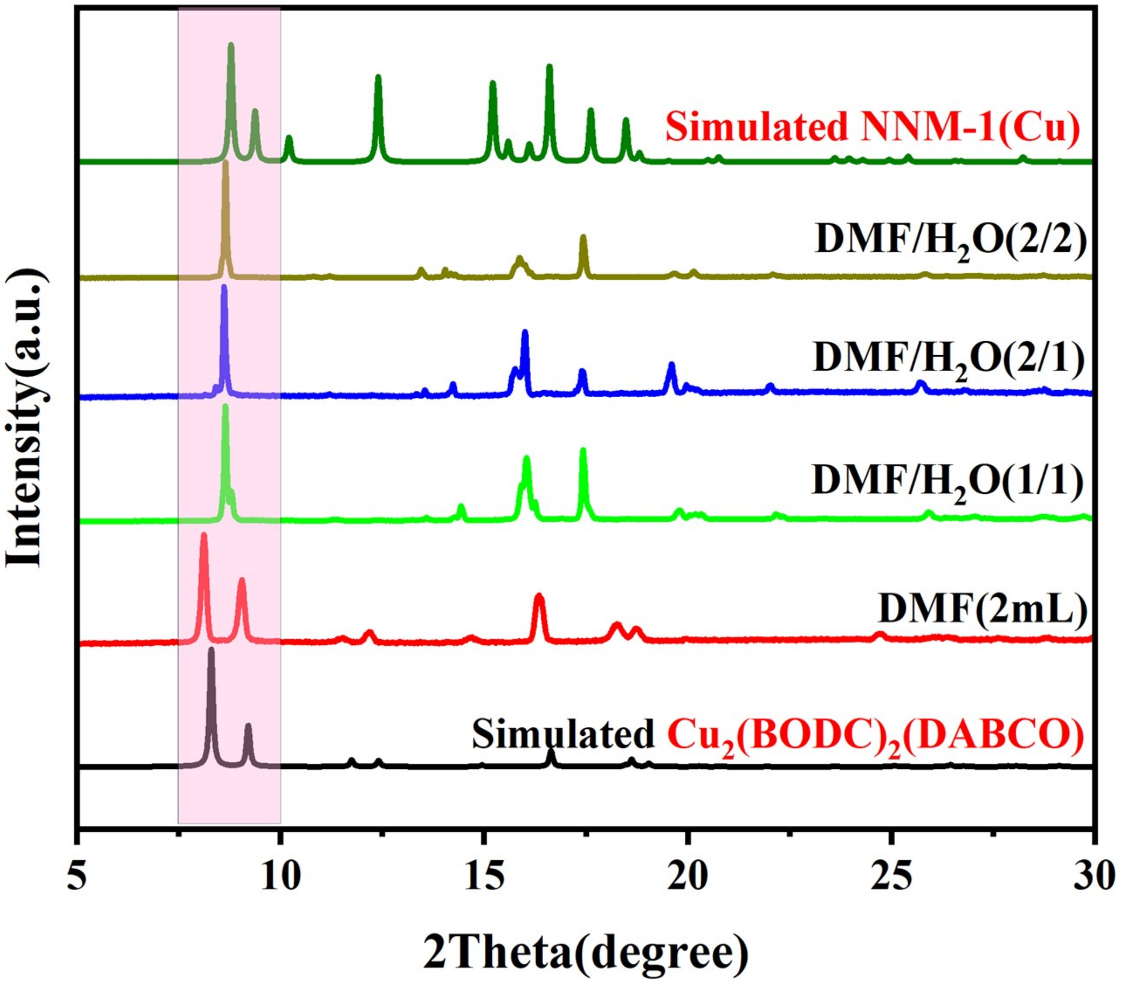

Figure S8. XRD comparisons of samples obtained by varying the solvent ratio of DMF/H_2O according to metal to ligands ratio in **$Cu_2(BODC)_2(DABCO)$** (that is Cu: BODC: DABCO = 0.18: 0.144: 0.116, reduced by five times the original scale).

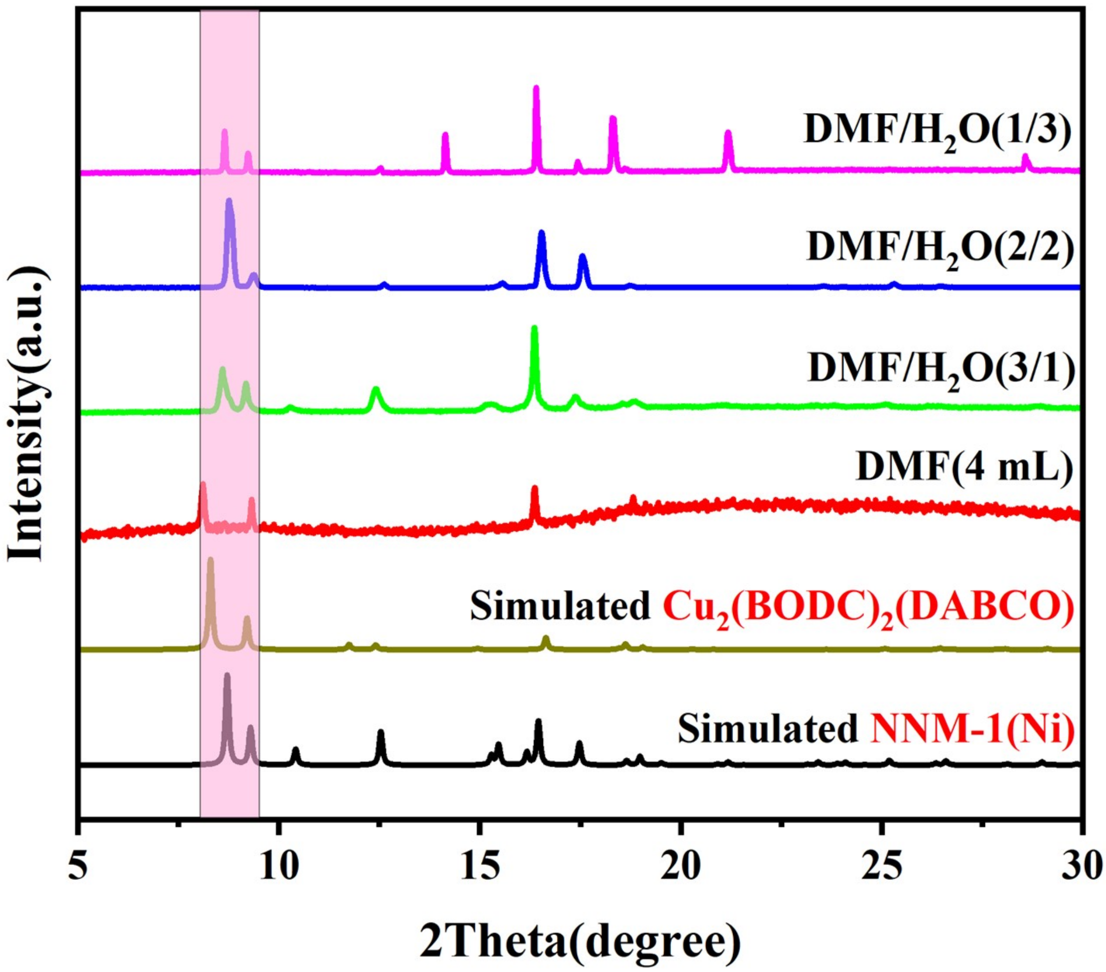

Figure S9. XRD comparisons of samples obtained by varying the solvent ratio of DMF/H_2O according to metal to ligands ratio in **NNM-1(Ni)** (that is Ni: BODC: DABCO = 0.045: 0.036: 0.029).

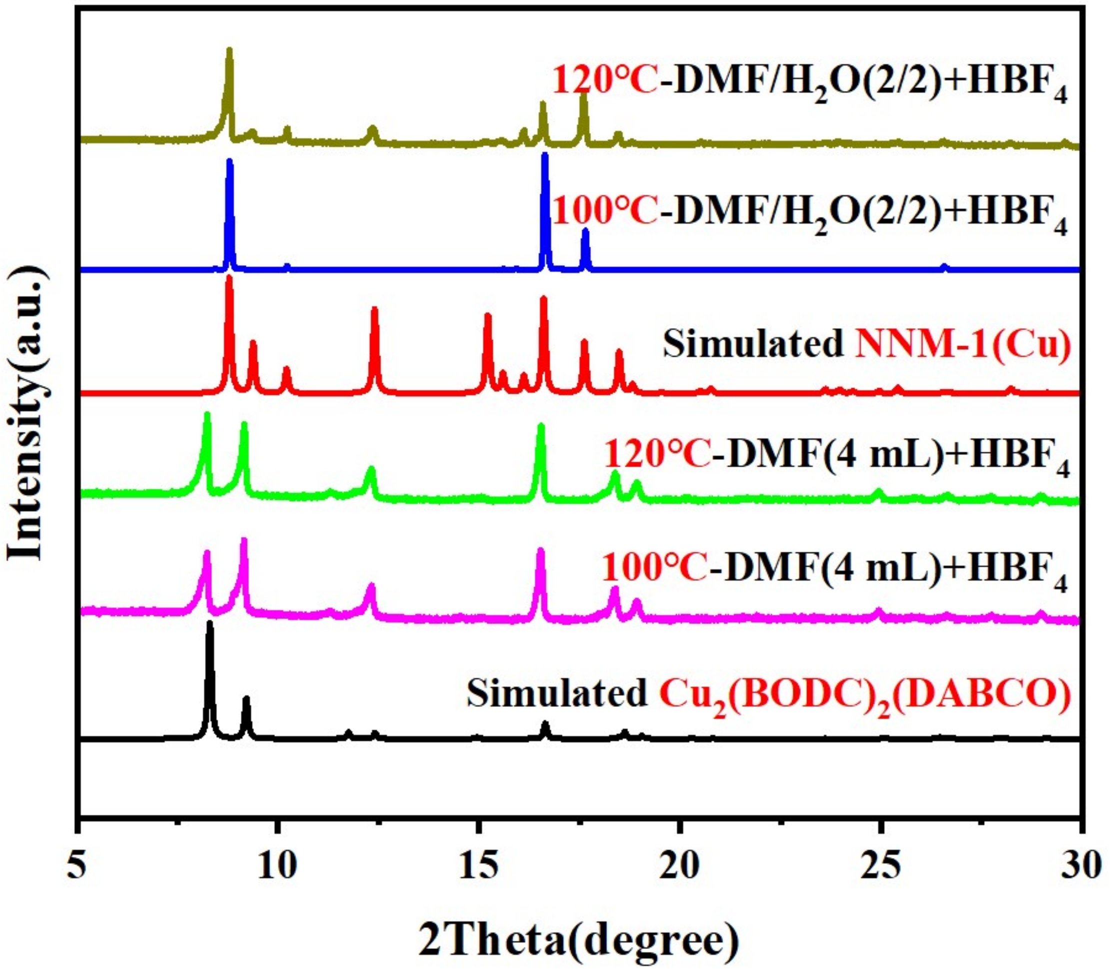

Figure S10. XRD comparisons of samples obtained by varying the solvent ratio of DMF/H_2O and reaction temperature according to experimental metal to ligands ratio in **NNM-1(Cu)** (that is Cu: BODC: DABCO = 0.045: 0.036: 0.029).

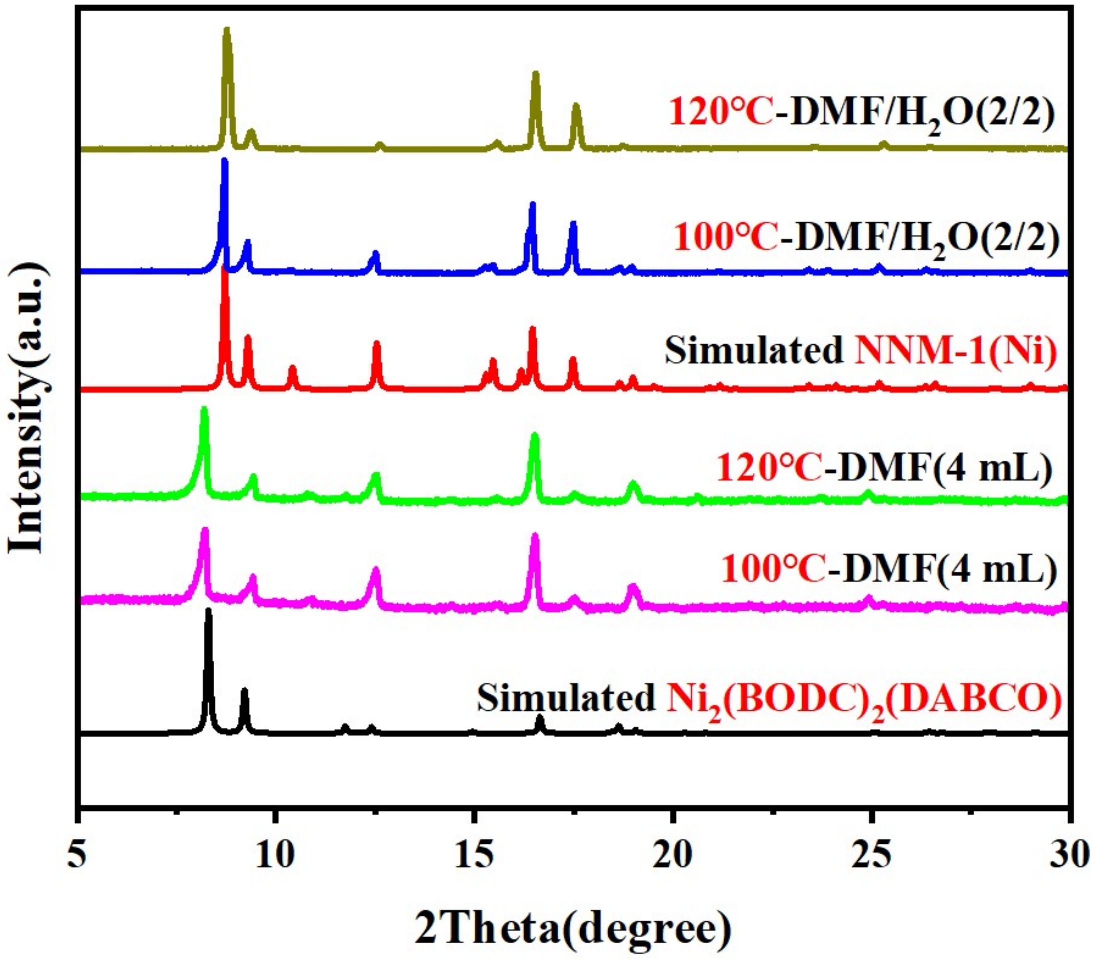

Figure S11. XRD comparisons of samples obtained by varying the solvent ratio of DMF/H_2O and reaction temperature according to metal to ligands ratio in **NNM-1(Ni)** (that is Ni: BODC: DABCO = 1: 2: 1).

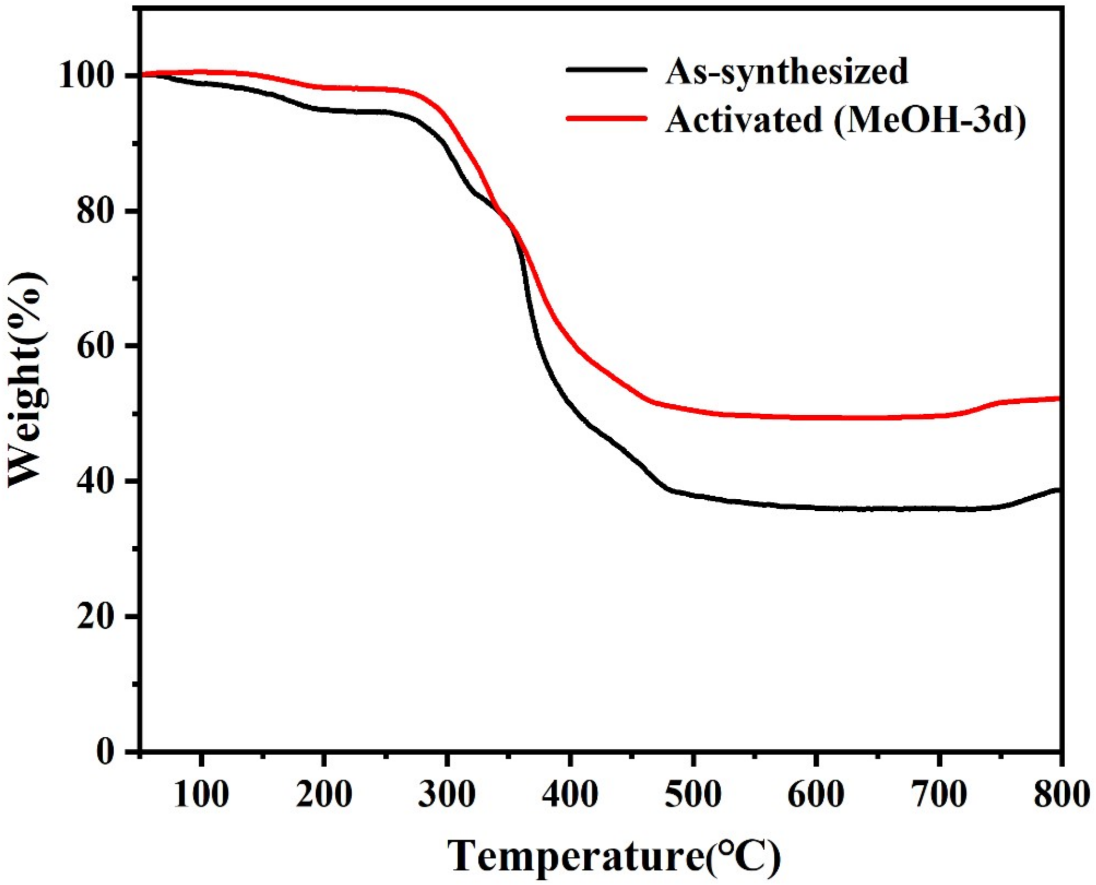

Figure S12. TGA curves of as-synthesized and activated **NNM-1(Cu)**.

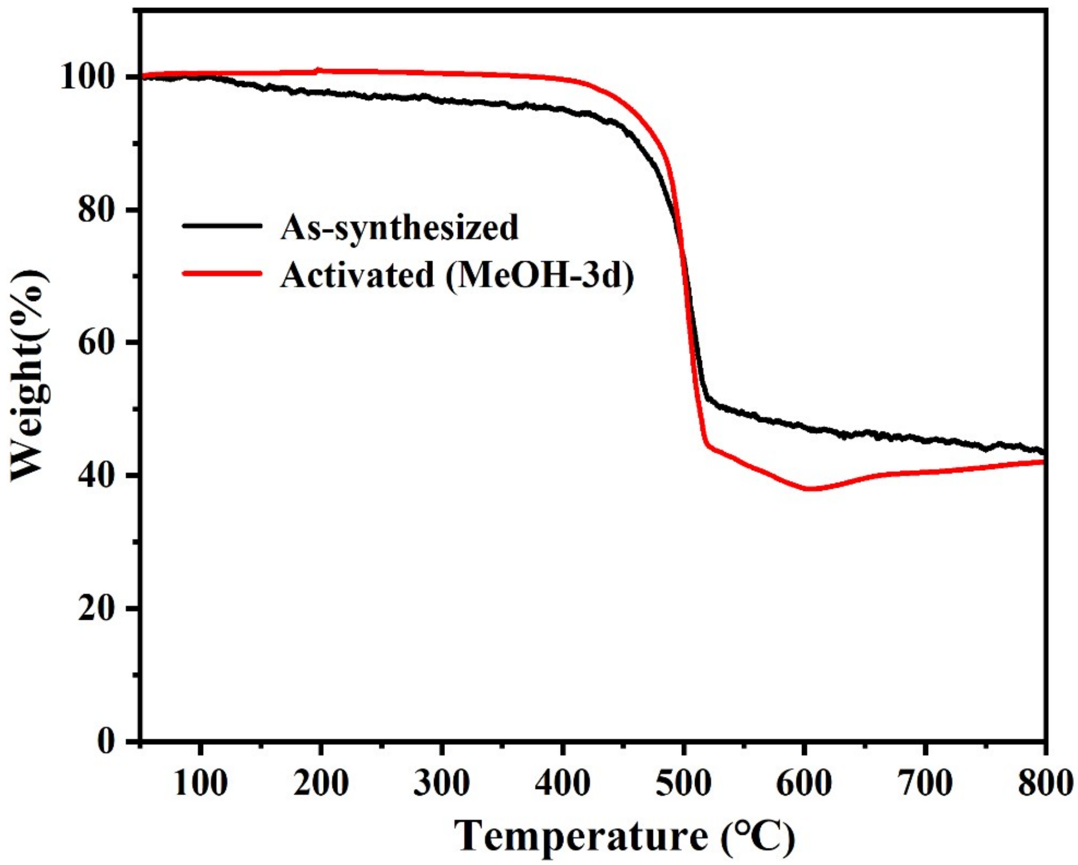

Figure S13. TGA curves of as-synthesized and activated **NNM-1(Ni)**.

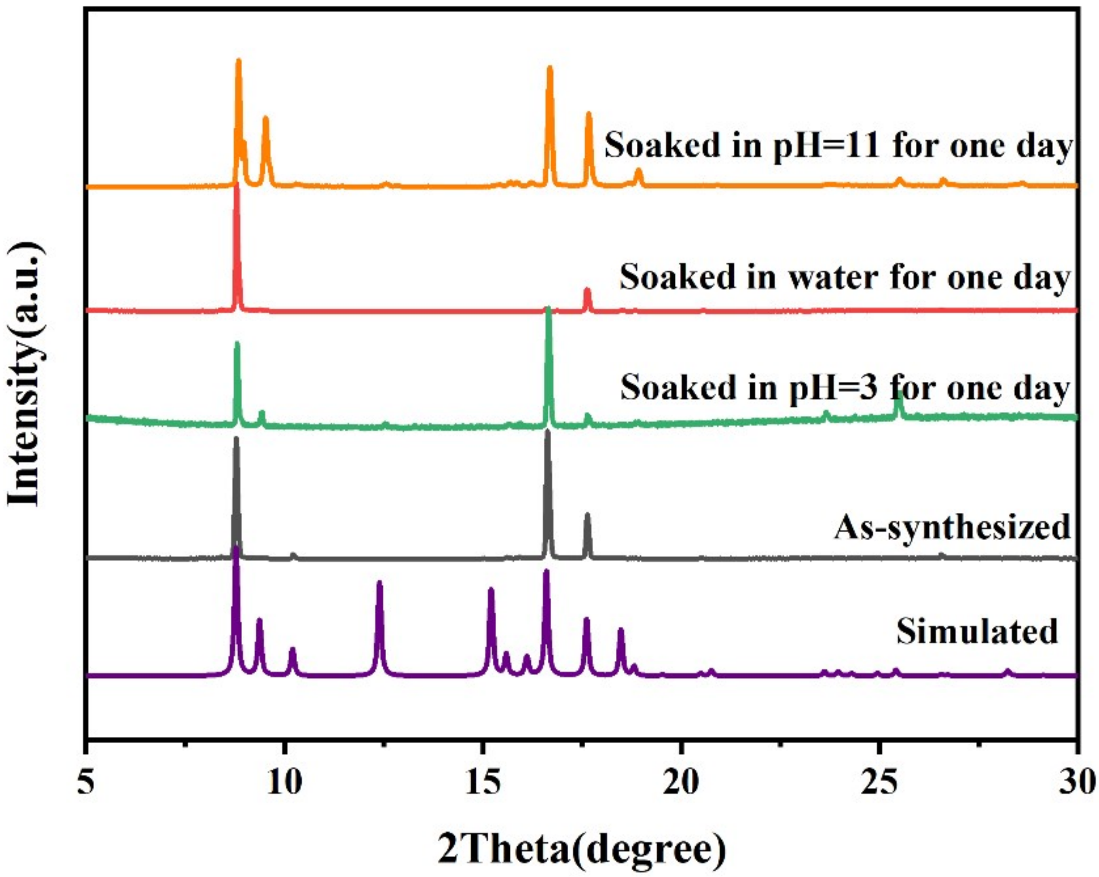

Figure S14. PXRD patterns of samples of **NNM-1(Cu)** treated with different pH conditions.

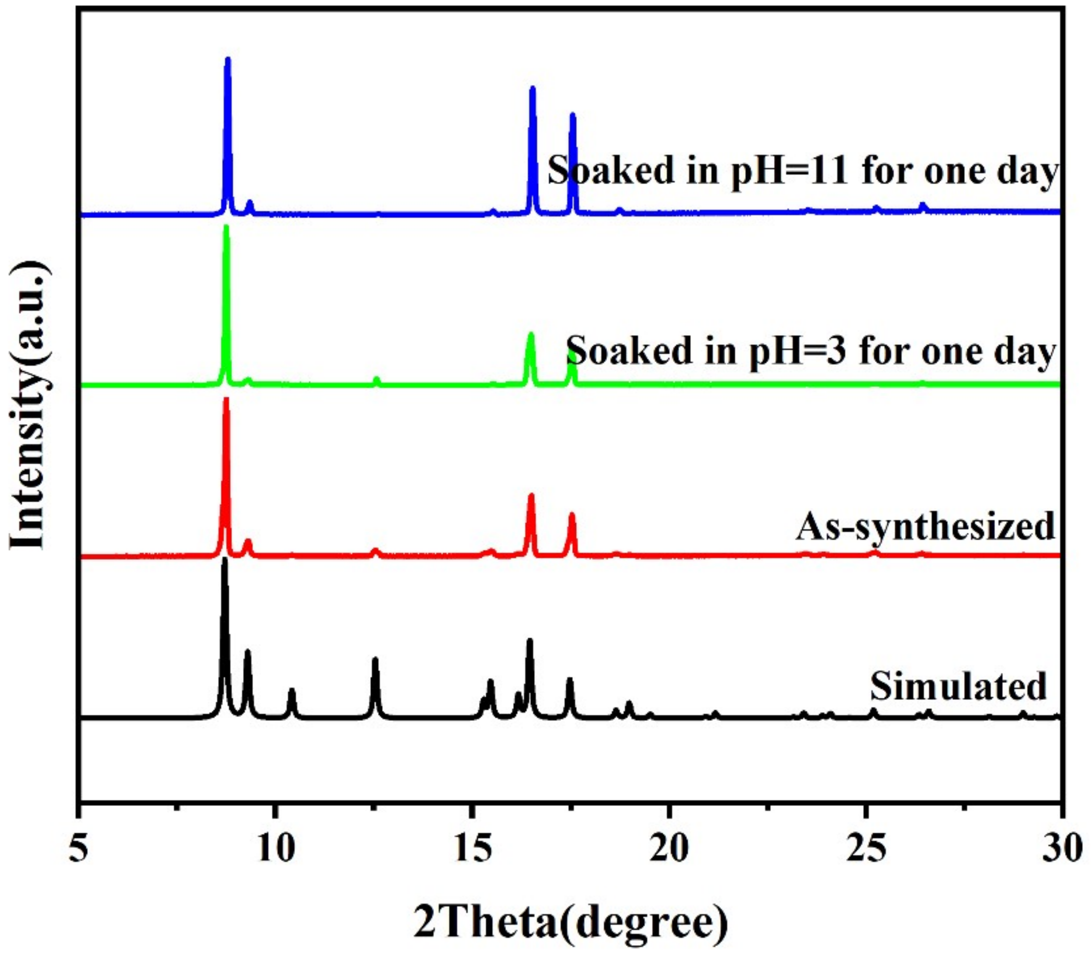

Figure S15. PXRD patterns of samples of **NNM-1(Ni)** treated with different pH conditions.

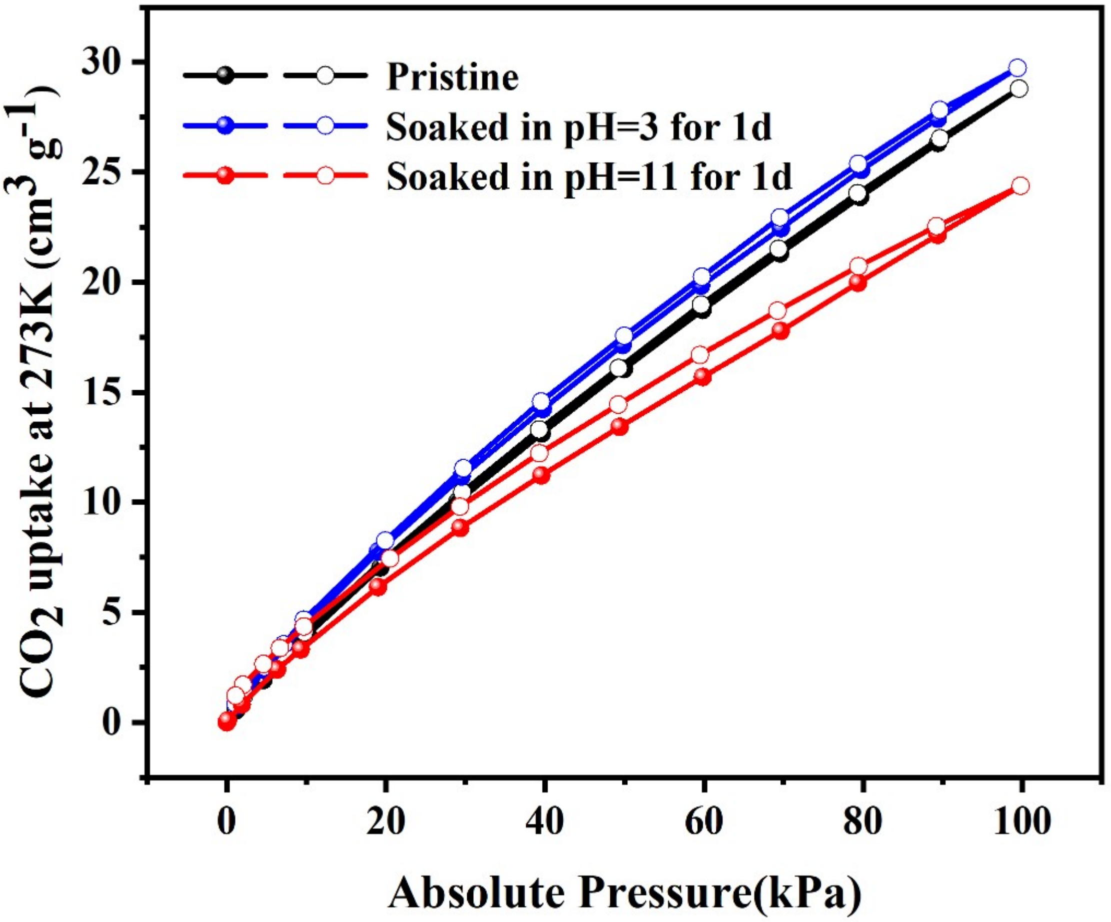

Figure S16. CO_2 adsorption and desorption isotherms at 273 K of **NNM-1(Cu)** treated with different pH conditions.

.

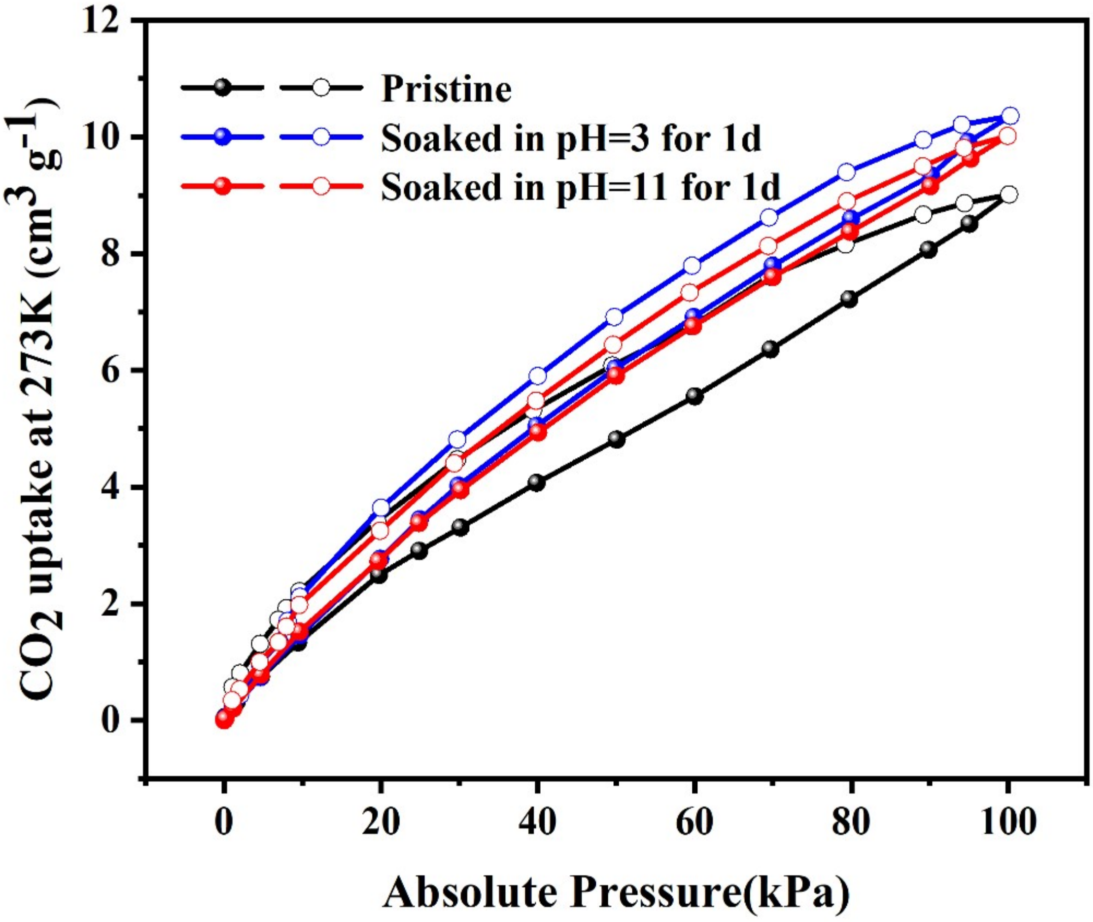

Figure S17. CO_2 adsorption and desorption isotherms at 273 K of **NNM-1(Ni)** treated with different pH conditions.

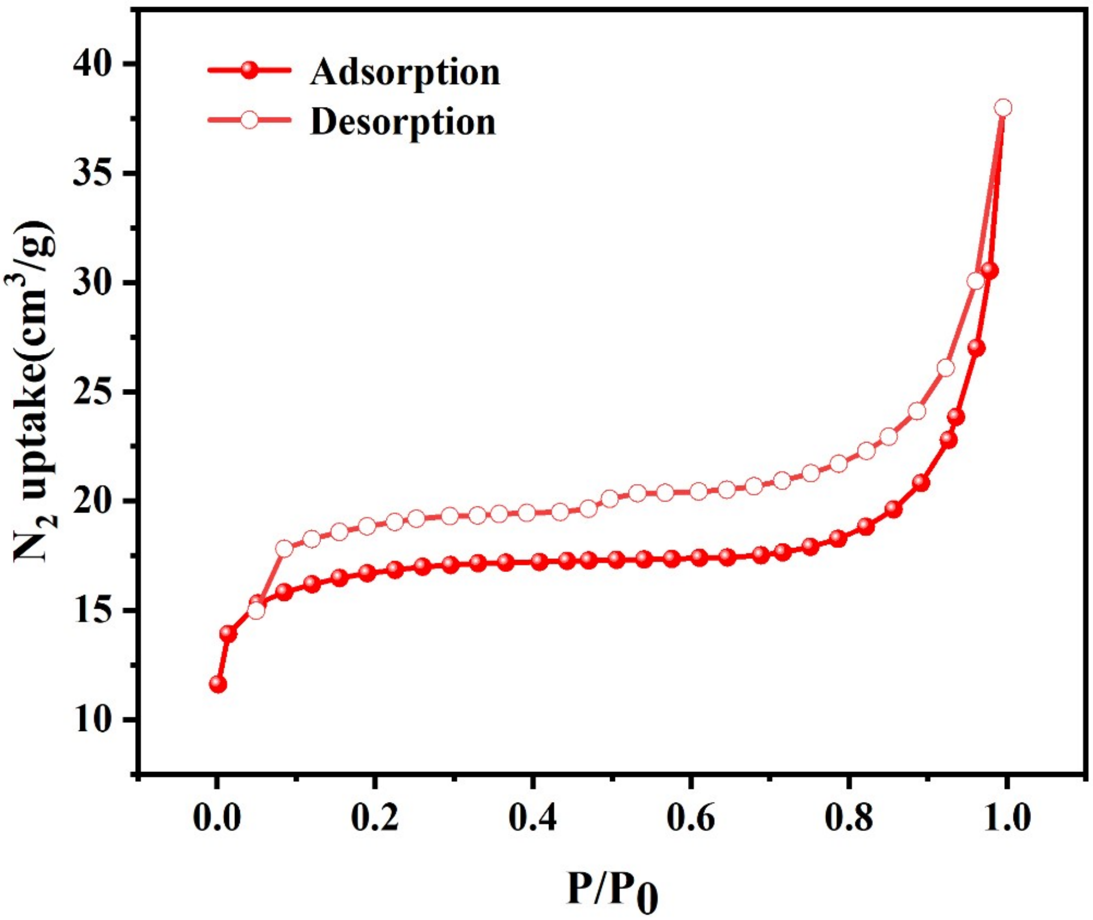

Figure S18. N_2 adsorption and desorption isotherms at 77 K of **NNM-1(Cu)**.

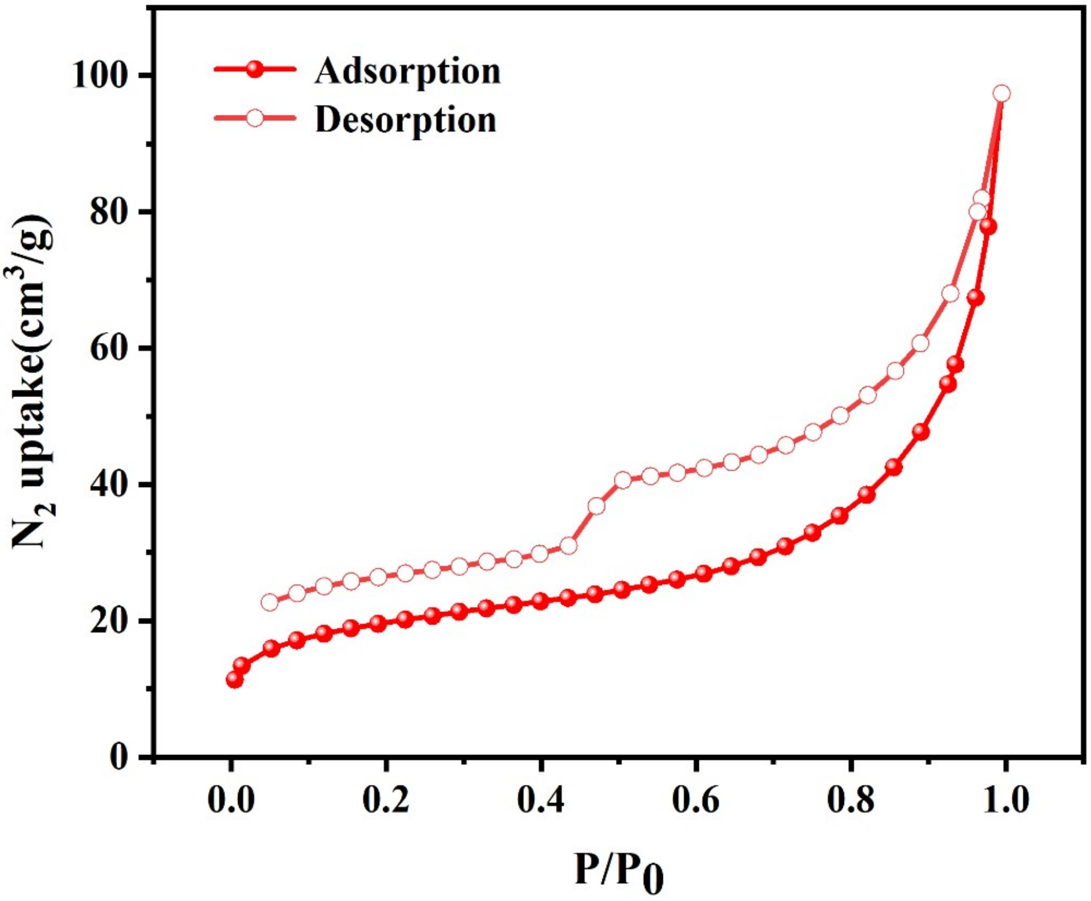

Figure S19. N_2 adsorption and desorption isotherms at 77 K of **NNM-1(Ni)**.

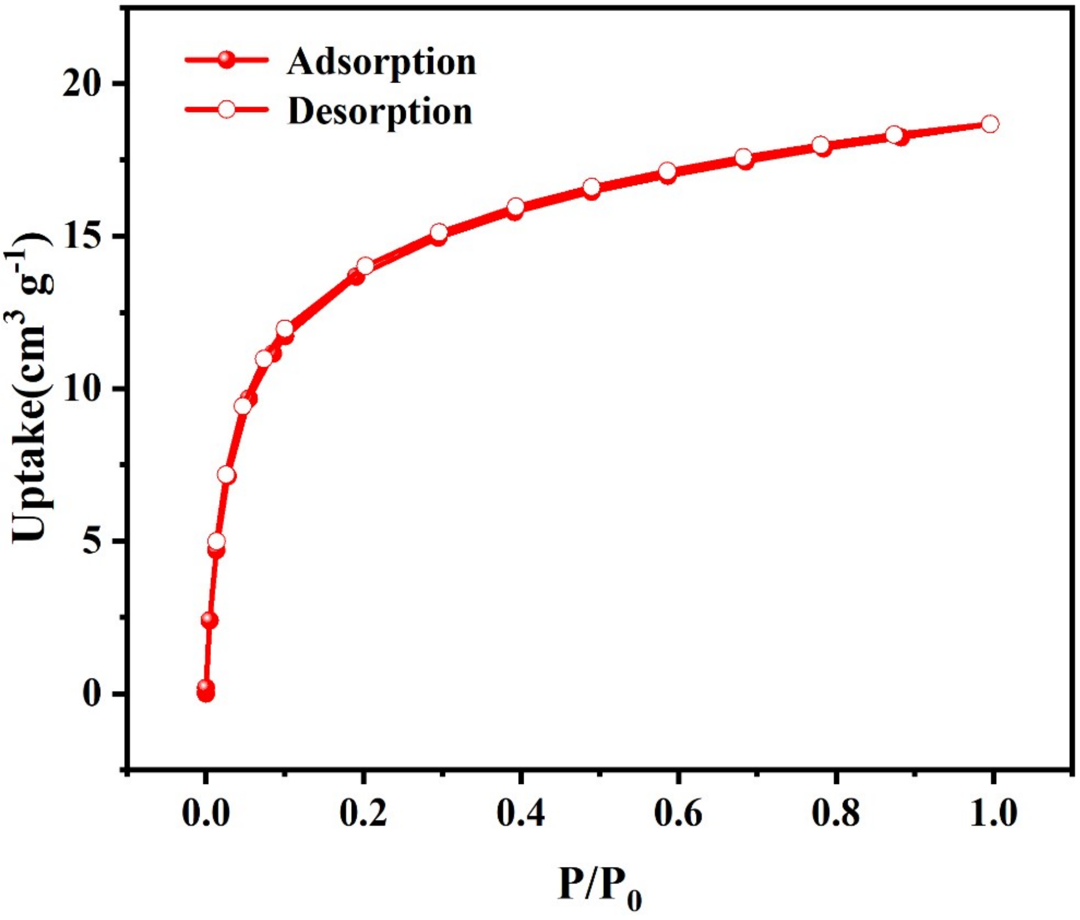

Figure S20. CO_2 adsorption and desorption isotherms at 195 K of **NNM-1(Cu)**.

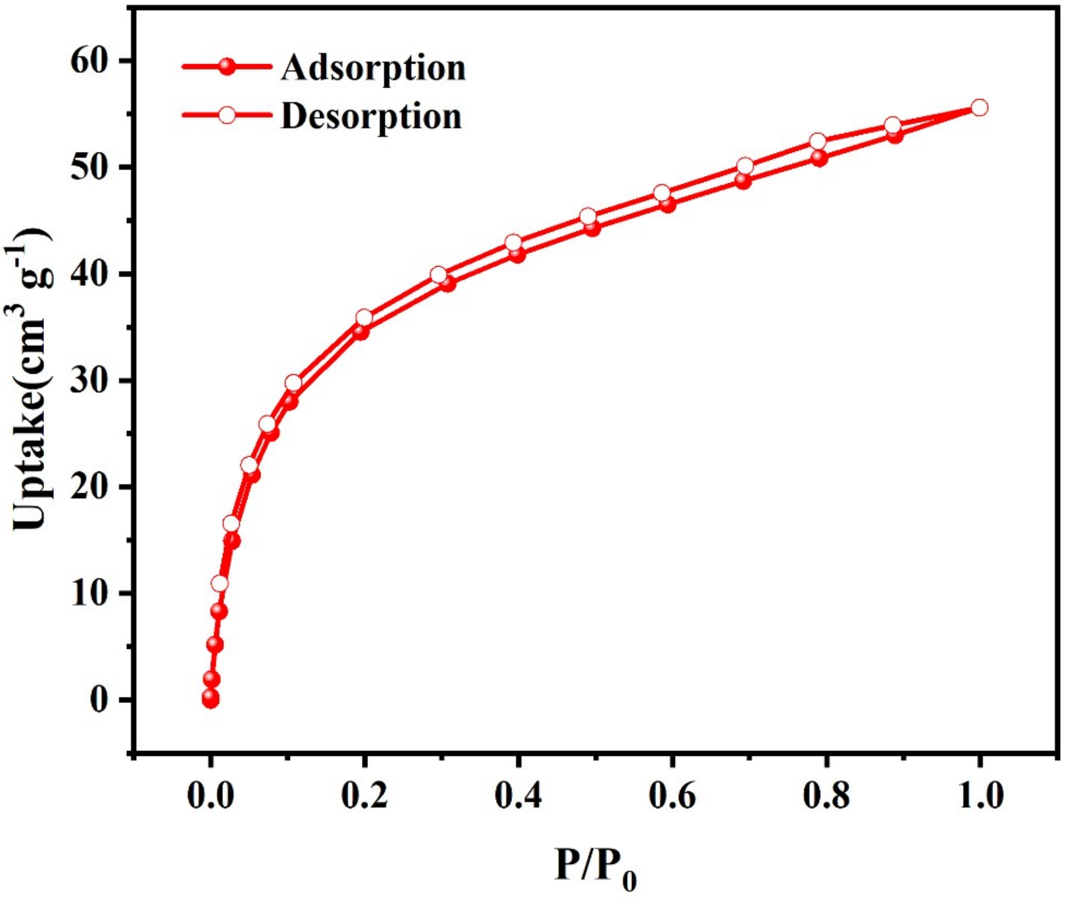

Figure S21. CO_2 adsorption and desorption isotherms at 195 K of **NNM-1(Ni)**.

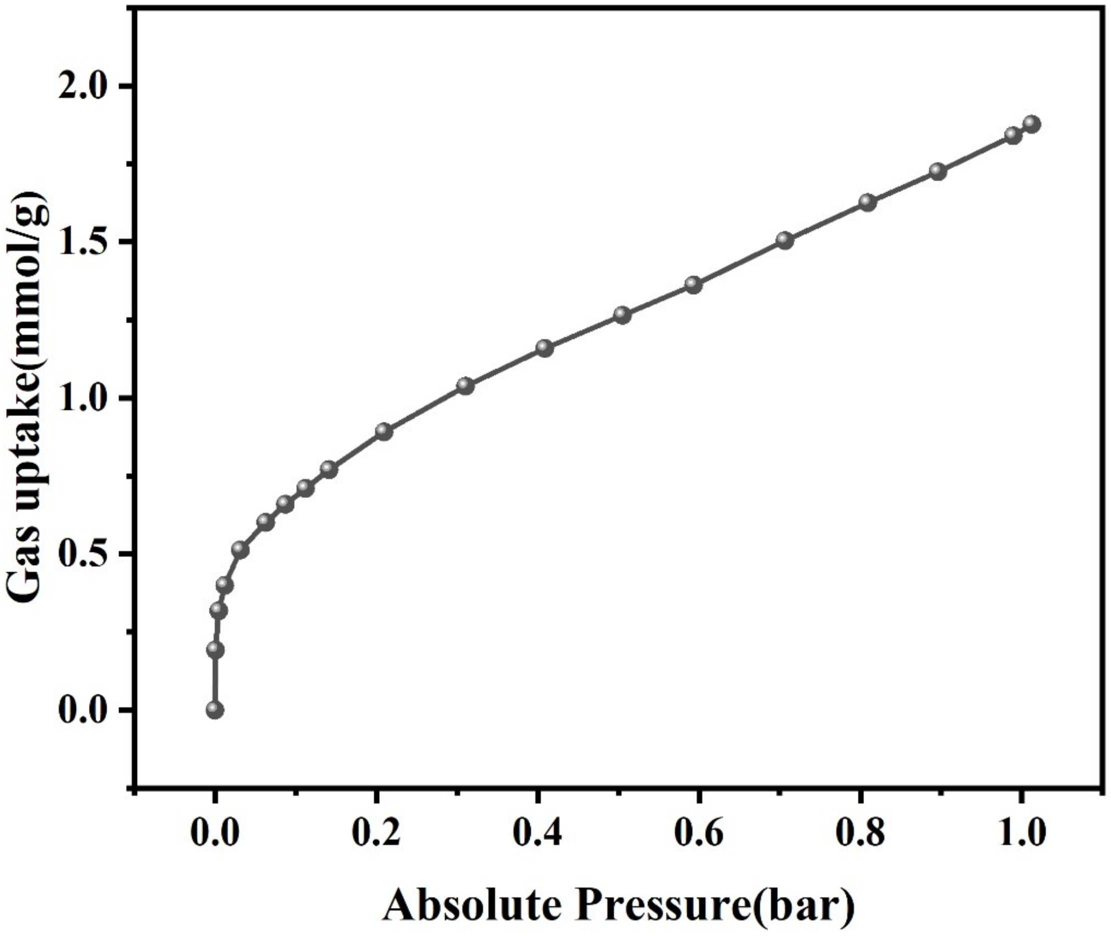

Figure S22. NH_3 adsorption isotherm of **NNM-1(Ni)** at 298 K.

.

Table S2. Comparison of NH_3 capture properties with some reported materials.

Material	Uptake (mmol/g)		Crystallographic density (g/cm^3)	Uptake (mg/cm^3)		Ref
	25 mbar	1 bar		25 mbar	1 bar	
**NNM-1(Cu)**	2.81	7.89	1.77	84.55	**237.4**	This work
**FDU-HOF-3**	8.1	9.34	1.335	183.8	212.0	1
**KUF-1a**	0.5	6.67	1.258	10.69	142.6	2
**HOF-102**	0.45	11.16	0.67	5.126	127.1	3
**HOF-101**	3.84	8.44	0.741	48.37	106.3	4

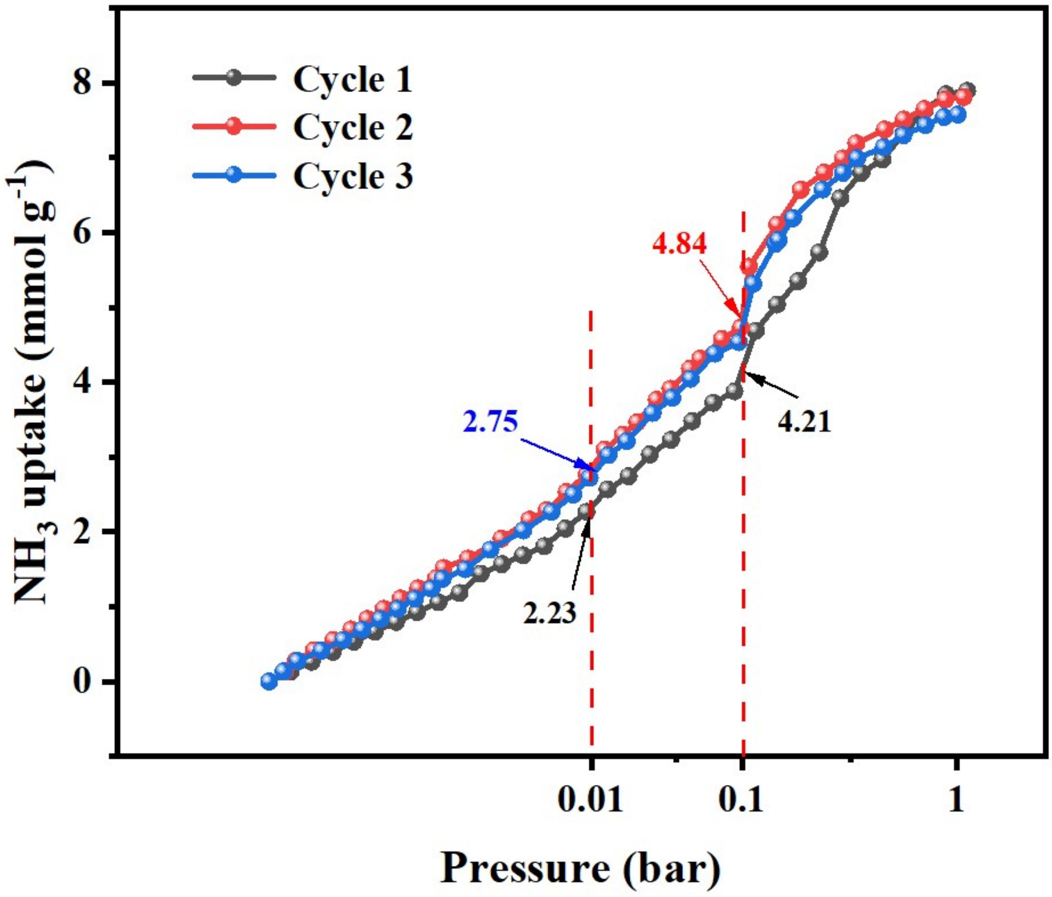

Figure S23. Three cycles of NH_3 adsorption isotherms of **NNM-1(Cu)** at 298 K.

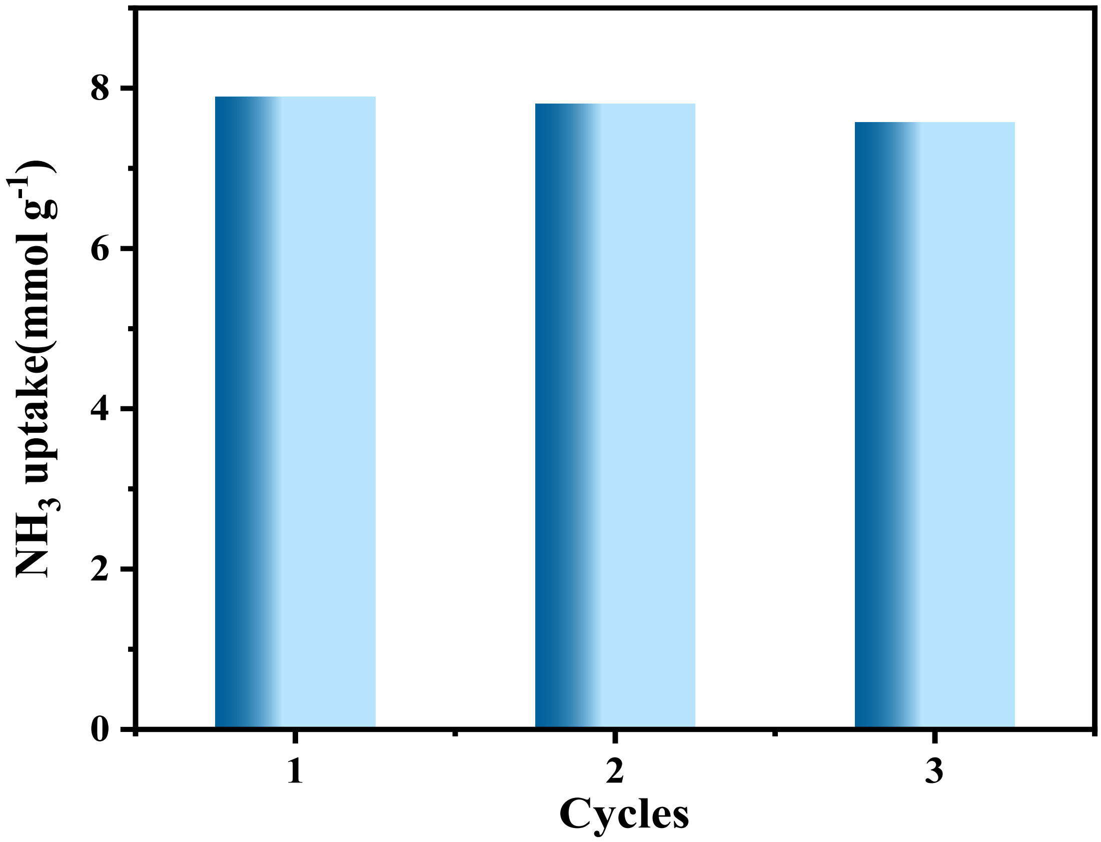

Figure S24. Comparison of the maximum NH_3 adsorption capacity of the three cycles of **NNM-1(Cu)** at 298 K.

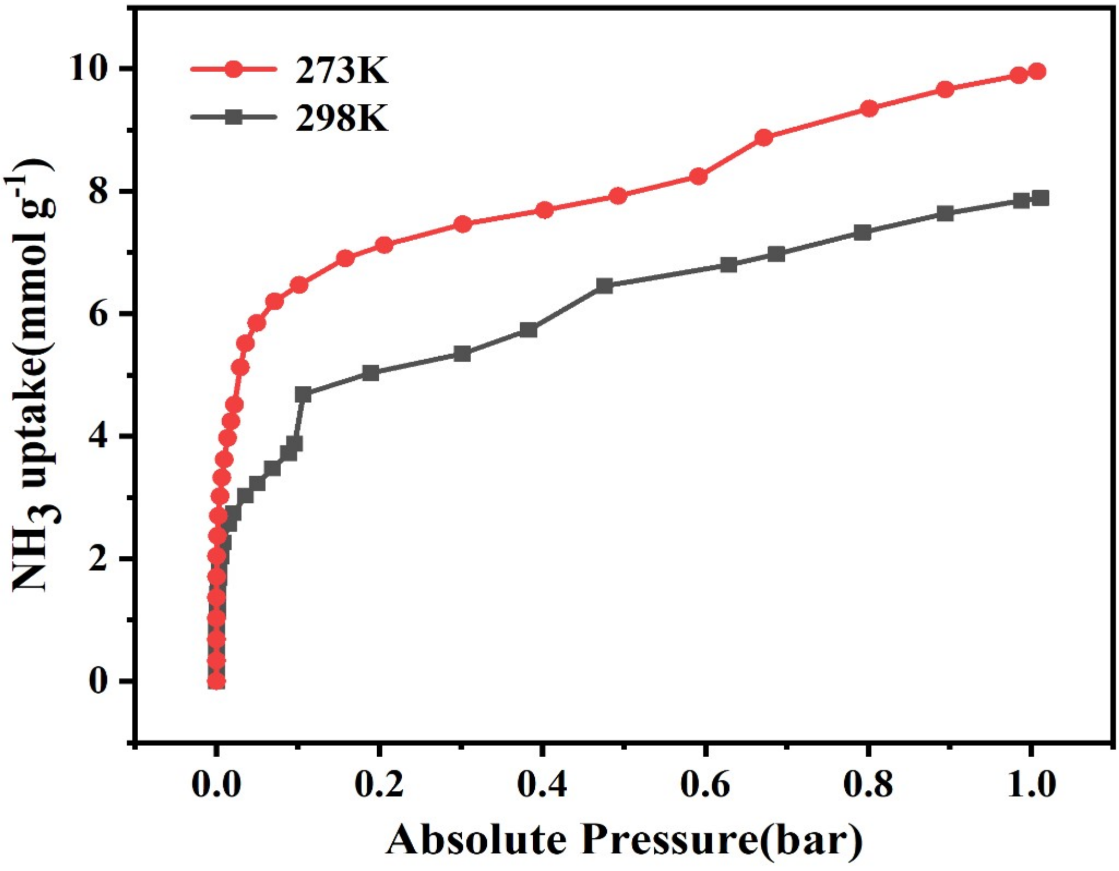

Figure S25. Comparison of NH_3 adsorption isotherms of **NNM-1(Cu)** at 273 K and 298 K.

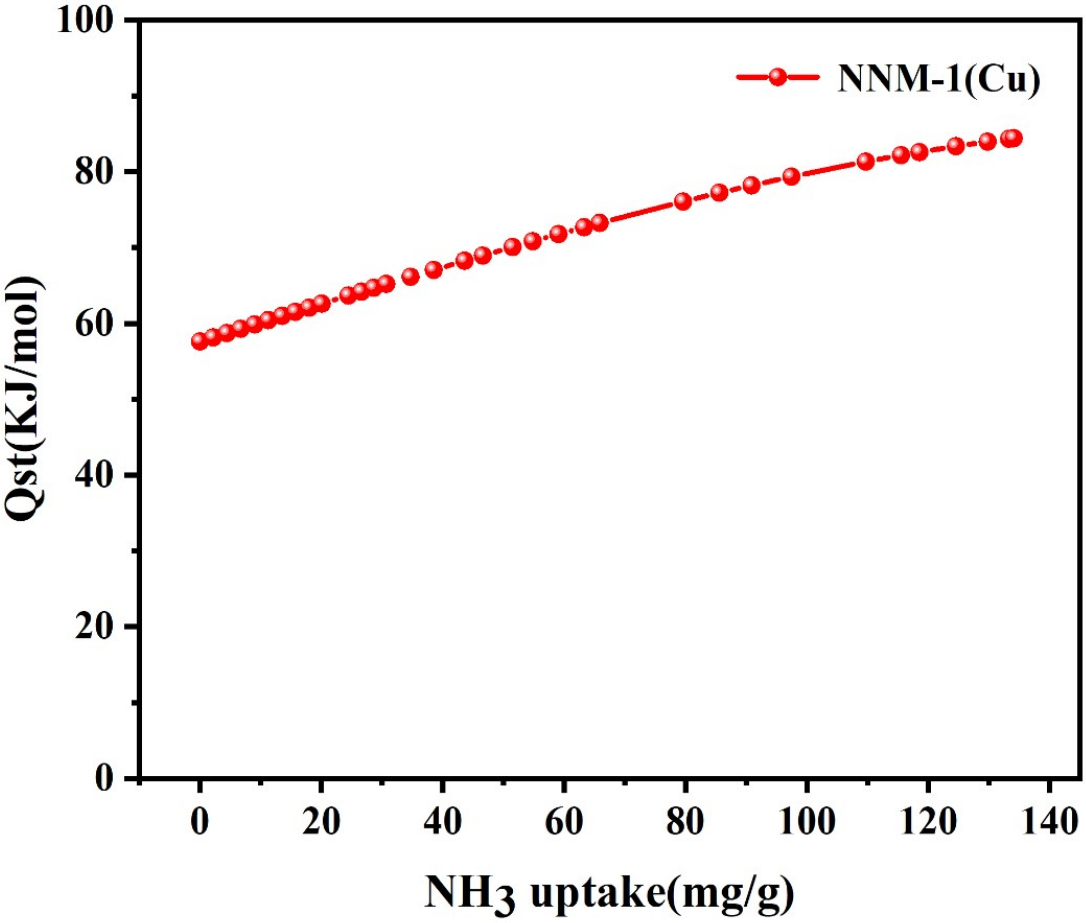

Figure S26. The isosteric heat of adsorption (Qst) of NH_3 for **NNM-1(Cu)**.

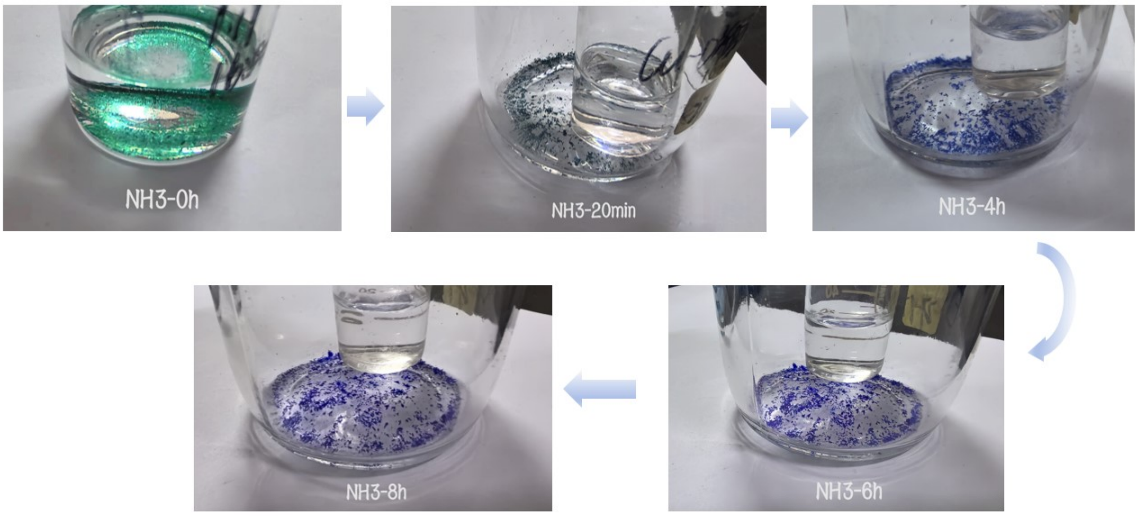

Figure S27. Monitoring process of NH_3 adsorption (adsorption of volatilization method, placing a vial filled with ammonia into a large vial with the bottom lined with the sample).

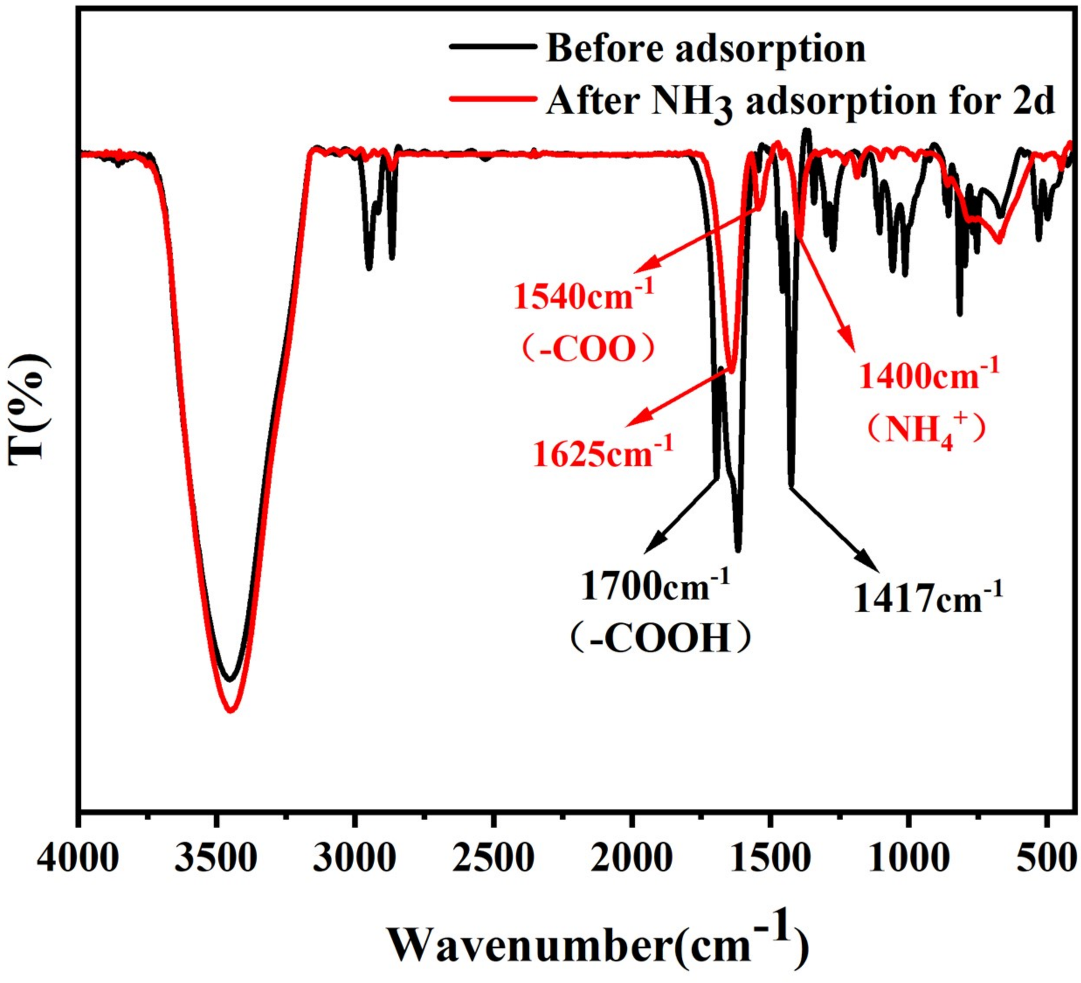

Figure S28. Comparison of the IR spectrum before and after NH_3 adsorption of **NNM-1(Cu)**.

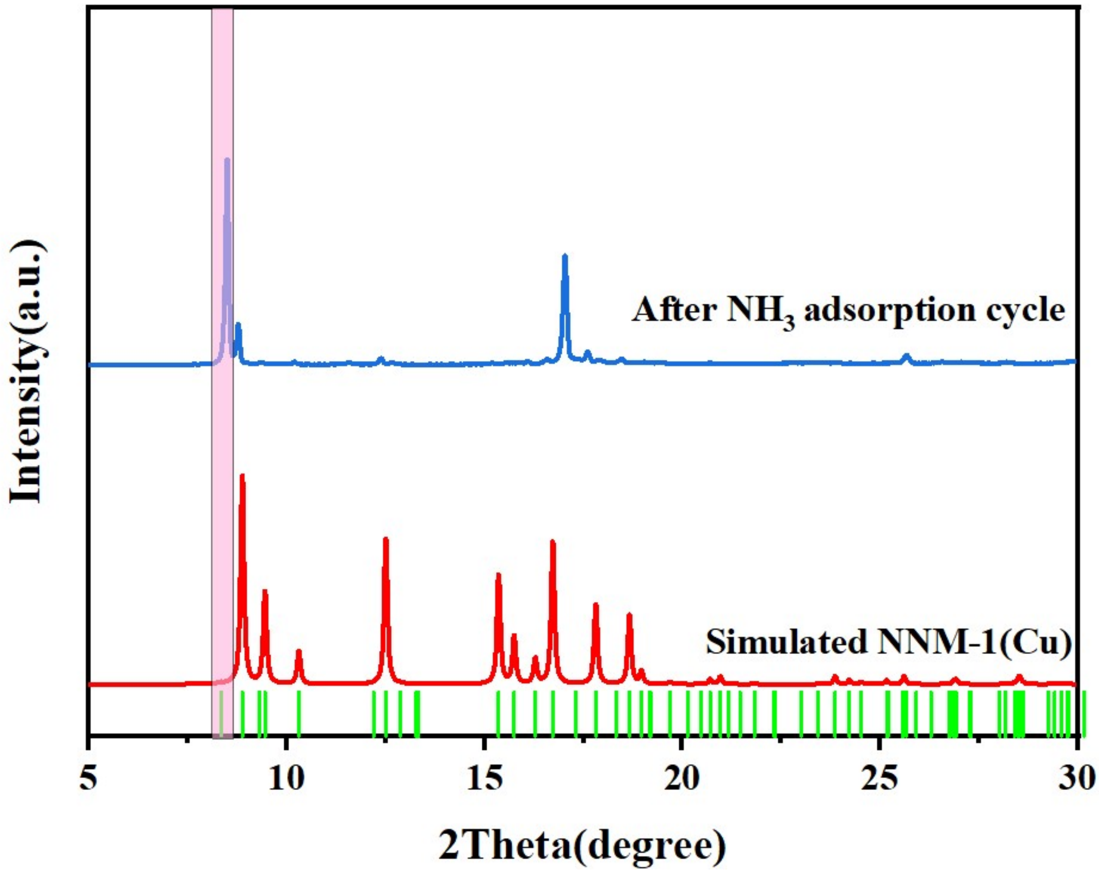

Figure S29. Comparisons of PXRD pattern of sample after testing the ammonia adsorption cycle with simulated PXRD patterns of **NNM-1(Cu)**.

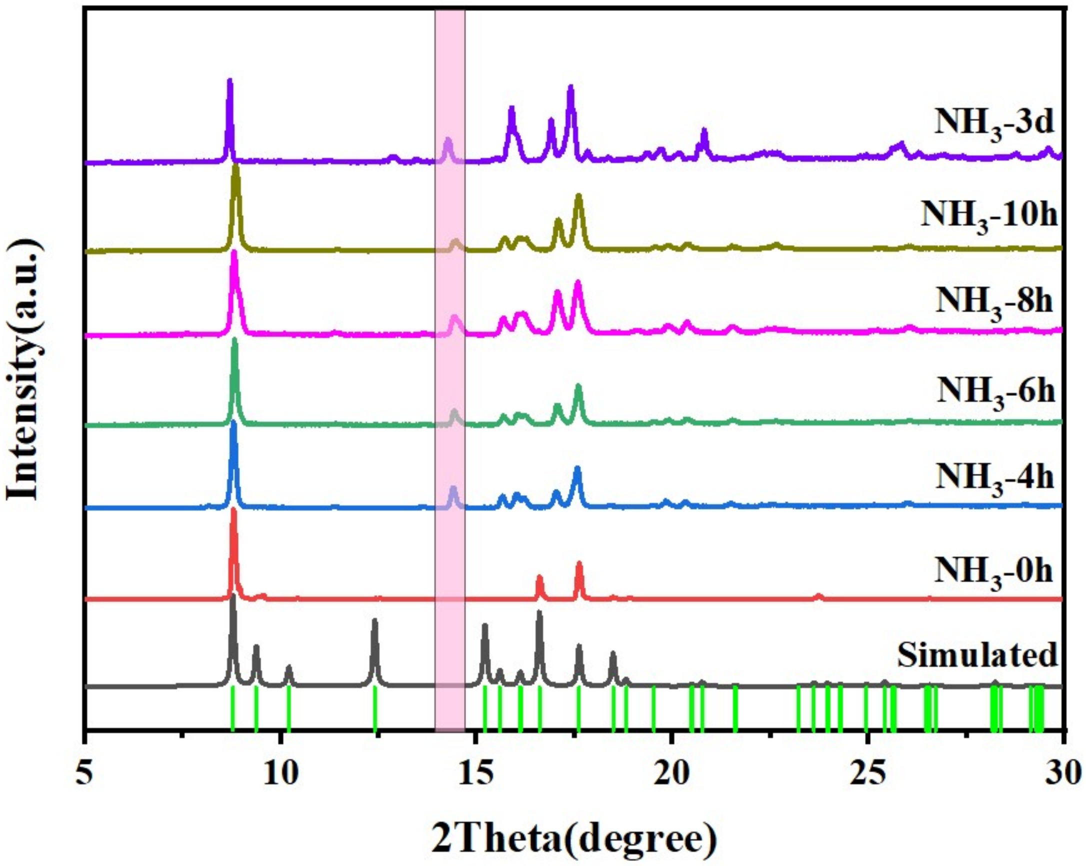

Figure S30. The variation of PXRD patterns between the process of NH_3 diffusion experiments of **NNM-1(Cu)**.

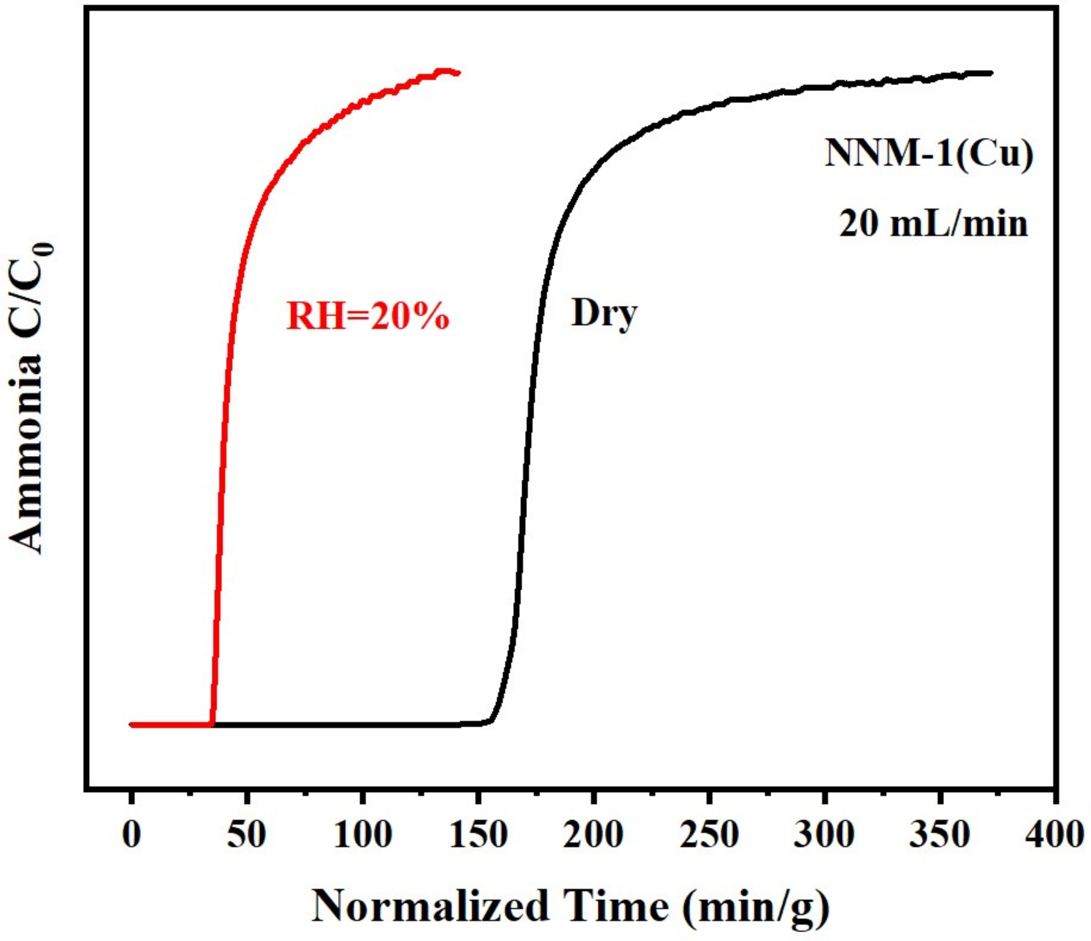

Figure S31. The breakthrough curves of NH_3 for **NNM-1(Cu)** under dry (black) and humid (20% RH, red) at a feed concentration of 1000 ppm.

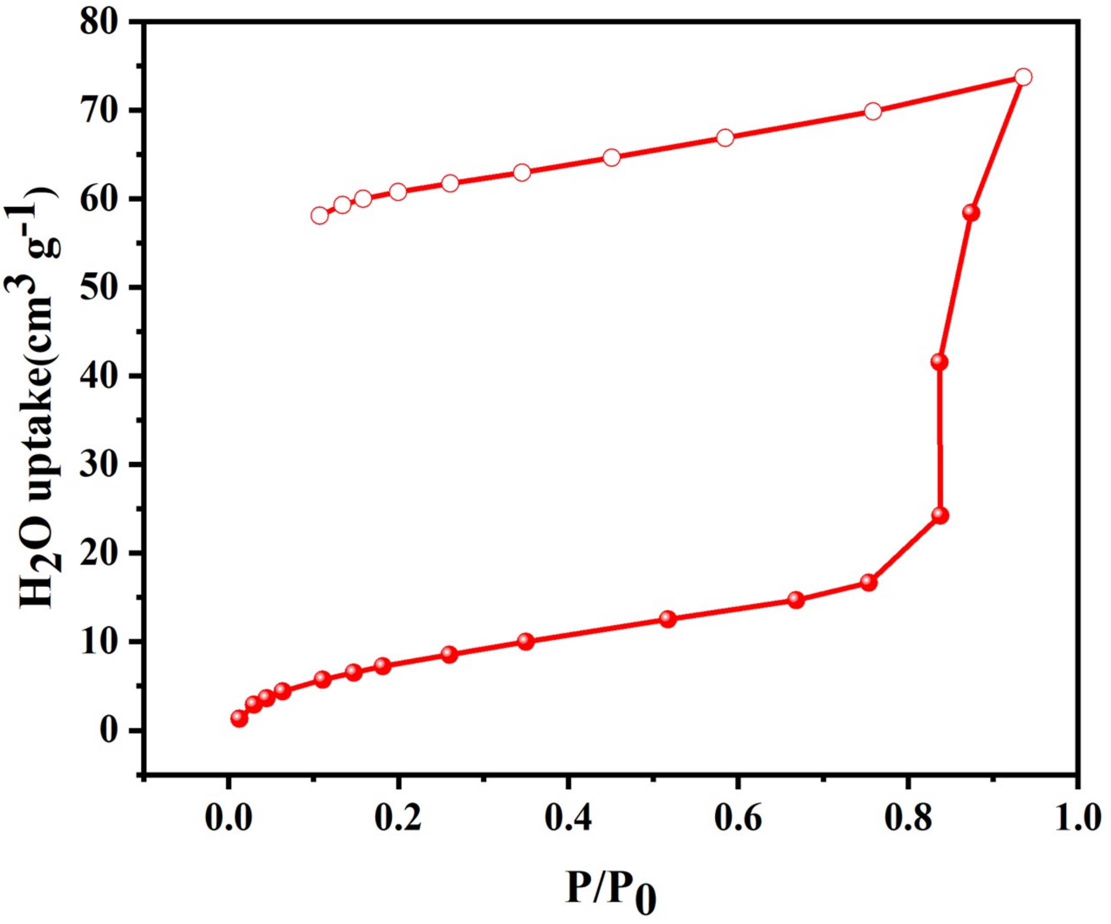

Figure S32. Water adsorption and desorption isotherms of **NNM-1(Cu)** at 298 K.

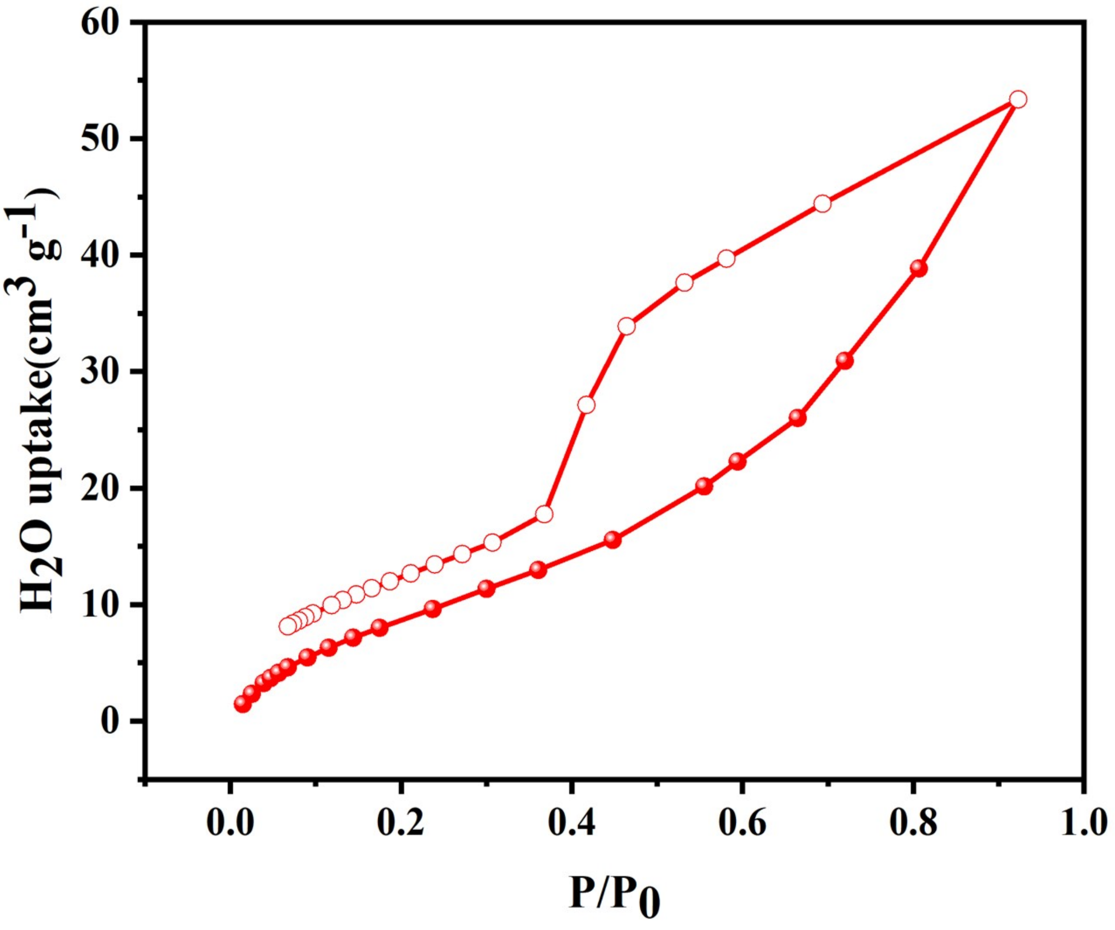

Figure S33. Water adsorption and desorption isotherms of **NNM-1(Ni)** at 298 K.
